# GRK5 regulates endocytosis of FPR2 independent of β-arrestins

**DOI:** 10.1016/j.jbc.2024.108112

**Published:** 2024-12-18

**Authors:** Christine E. Jack, Emily M. Cope, Laura Lemel, Meritxell Canals, Julia Drube, Carsten Hoffmann, Asuka Inoue, James N. Hislop, Dawn Thompson

**Affiliations:** 1 School of Medicine, Medical Sciences and Nutrition, Institute of Medical Sciences, University of Aberdeen, United Kingdom; 2 Division of Physiology, Pharmacology and Neuroscience, School of Life Sciences, University of Nottingham, Nottingham, United Kingdom; 3 Institut für Molekulare Zellbiologie, CMB—Center for Molecular Biomedicine, Universitätsklinikum Jena, Friedrich-Schiller-Universität Jena, Jena, Germany; 4 Graduate School of Pharmaceutical Sciences, Tohoku University, Sendai, Miyagi, Japan; 5 Graduate School of Pharmaceutical Sciences, Kyoto University, Kyoto, Japan

**Keywords:** GPCR, FPR2, GRK, arrestin, signaling, trafficking

## Abstract

The formyl-peptide receptor 2 (FPR2) is a G-protein–coupled receptor that responds to pathogen-derived peptides and regulates both proinflammatory and proresolution cellular processes. While ligand selectivity and G-protein signaling of FPR2 have been well characterized, molecular mechanisms controlling subsequent events such as endocytosis and recycling to the plasma membrane are less understood. Here, we show the key role of the G-protein–coupled receptor kinase 5 (GRK5) in facilitating FPR2 endocytosis and postendocytic trafficking. We found, in response to activation by a synthetic peptide WKYMVm, the recruitment of β-arrestins to the receptor requires both putative phosphorylation sites in the C-terminal region of FPR2 and the presence of GRKs, predominantly GRK5. Furthermore, although GRKs are required for β-arrestin recruitment and endocytosis, the recruitment of β-arrestin is not itself essential for FPR2 endocytosis. Instead, β-arrestin determines postendocytic delivery of FPR2 to subcellular compartments and subsequent plasma membrane delivery and controls the magnitude of downstream signal transduction. Collectively, the newly characterized FPR2 molecular pharmacology will facilitate the design of more efficient therapeutics targeting chronic inflammation.

Resolution pharmacology is a rapidly expanding field of research and essential for healing and efficient recovery in response to injury or infection (1). Central to this process is the formyl peptide receptor 2 (FPR2), an immune receptor and member of the G-protein–coupled receptor (GPCR) superfamily and a key driver in proresolution signaling (2). Previous research has shown disruption of FPR2 function, by either deletion of the receptor (3, 4) or its endogenous ligand (5), results in enhanced proinflammation, and has a detrimental impact on whole animal physiology. Furthermore, agonists at FPR2 have been beneficial for the treatment of chronic inflammation in preclinical studies (4, 6, 7, 8, 9, 10, 11, 12, 13). Hence, the ability to harness these responses clinically for the treatment of inflammatory disease would be the holy grail for the pharmaceutical industry with efforts currently focused on the development of new ligands targeting specific proresolving receptors or the development of endogenous mimetics.

Over the past decade, several compounds targeting FPR2 have been developed to treat the proinflammation that accompanies heart failure by driving resolution processes. Indeed, compounds synthesized by Actelion and Bristol Myers Squibb (ACT-389949 (14) and BMS-986235 (10) respectively) underwent phase I clinical trials; however, ACT-389949 failed to progress further because of a loss of efficacy following repeated exposure, initially attributed to receptor downregulation. To further elucidate the mechanism behind this “tolerance,” an elegant study by Lupisella *et al.* (15) assessed the molecular pharmacology *in vitro* and physiology *in vivo* of these two compounds and determined differences in endocytic trafficking as responsible for differences in efficacy of ligands in clinical trial. Thus, regulation of receptor function must be of critical importance because of the switch nature of physiological function. Hence, in order for new drugs to be designed, it is important to determine how this therapeutically vital receptor is regulated and how this translates to whole animal physiology.

It is well documented that once activated many GPCRs are rapidly phosphorylated by GPCR-regulated kinases (GRKs) to prevent further activation and, following the recruitment of β-arrestins, rapidly internalized into endosomes in a clathrin- and AP2-dependent manner (16). Previous studies have shown that GRK2 phosphorylation of the C-tail is required for internalization of the related FPR1, yet β-arrestin association is dispensable (17, 18, 19, 20, 21). Furthermore, we have previously shown a role for putative phosphorylation sites within the C-terminus for the recruitment of β-arrestins to the FPR2 (22); however, it has not been investigated which kinases control subsequent β-arrestin recruitment nor the requirement for endocytic trafficking and/or signal transduction. Given the reported importance internalization has on therapeutic efficacy, understanding how it is regulated is vital for the design of pathway-specific ligands. Here, we investigate the role GRKs have on β-arrestin recruitment, receptor internalization, and signaling of FPR2. We show that β-arrestin recruitment and internalization is GRK dependent, but β-arrestin is not an absolute requirement for endocytosis to occur. In addition, we have generated a β-arrestin-“biased” FPR2 and shown ablation of downstream signaling indicating that agonist-mediated signaling occurs exclusively *via* G_αi_. Importantly, we provide evidence for the first time that β-arrestin recruitment is important for postendocytic sorting of the FPR2 and controls the extent of recycling to the plasma membrane by delaying transit through intracellular compartments and thus impacts the magnitude of the pERK response.

## Results

### Mutation of serine and threonine clusters within the C-tail of FPR2 results in altered β-arrestin recruitment

**Figure 1:**
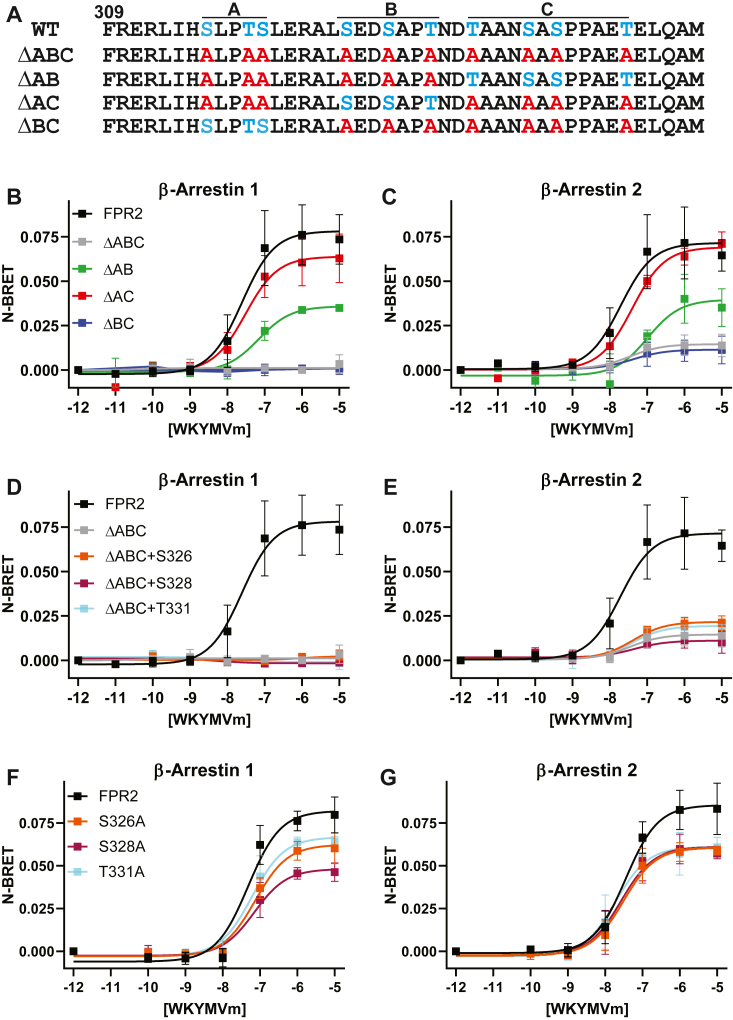
**Serine–threonine dependence of β-arrestin recruitment to FPR2.***A,* schematic of FPR2 C terminus and mutations investigated. BRET analysis of (*B*, *D*, *F*) β-arrestin 1 and (*C*, *E*, *G*) β-arrestin 2. HEK293 cells were transfected with FPR2 or FPR2 mutants fused C-terminally to RLuc8 along with β-arrestin 1 or 2 fused to Venus fluorescent protein. Cells were treated with indicated concentrations of WKYMVm for 10 min before measurement. Data are expressed as the normalized luminescence of acceptor:donor, and the mean ± SD of at least three independent experiments was performed in triplicate. BRET, bioluminescence resonance energy transfer; FPR2, formyl-peptide receptor 2; HEK293, human embryonic kidney 293 cell line.

Following receptor activation, the canonical model for GPCR regulation involves the phosphorylation of serine and threonine residues within intracellular domains by GRKs, which promotes association with β-arrestins 1 and 2, followed by recruitment to clathrin-coated pits (CCPs) and subsequent internalization (16). In an attempt to characterize the regulation of FPR2, we first investigated the role of C-terminal putative phosphorylation sites for β-arrestin recruitment. Our previous studies have demonstrated that the FPR2 visibly recruits both β-arrestin 1 and 2, where it remains associated within endosomal compartments, a phenotype previously observed with other rhodopsin family GPCRs, often referred to as a class B phenotype (22). Consistent with this, we have further shown endosomal recruitment is controlled by a serine–threonine cluster (SXXSXXT), designated B, within the C-terminal tail (Fig. 1*A*). Here, we extended those studies to quantify the recruitment of β-arrestin 1 and 2 to the FPR2 using bioluminescence resonance energy transfer (BRET)–based interaction studies. We generated a C-terminal fusion of our previously described N-terminal FLAG-tagged FPR2 (22) with Renilla luciferase (FPR2-Rluc8), which robustly recruited both β-arrestins 1 and 2, following treatment with the synthetic peptide agonist WKYMVm (Fig. 1*B* and *C*). In addition to the previously described ΔAB mutant (22), we generated luciferase fusions of three further C-tail mutants, ΔABC, ΔAC, and ΔBC, where serine and threonine residues in these C-terminal regions were mutated to alanine (Fig. 1*A*). Mutation of all C-terminal serine and threonine residues to alanine (ΔABC) completely prevented the recruitment of either β-arrestin 1 and β-arrestin 2 to the receptor following agonist stimulation (Fig. 1*B* and *C*, Table 1). However, G-protein interaction was unaffected, and ΔABC displayed comparable Emax to WT FPR2 (Suppl. Fig. S1, *A and B*, *p* = 0.8505). Our previous findings reported that serines and threonines in cluster B of the C-tail were necessary for β-arrestin recruitment as deletion of ΔB or ΔAB putative phosphorylation sites partially inhibited endosomal colocalization (22). In agreement with these findings, the ΔAB mutant resulted in a ∼50% reduction in Emax (Fig. 1*A* and *B*, Table 1), whereas ΔAC exhibited either minimal or no impact on recruitment to the receptor (Fig. 1*B* and *C*, Table 1). In contrast, ΔBC almost completely abolished the ability of this mutant to recruit either β-arrestin 1 or β-arrestin 2. We further interrogated the importance of this B cluster further by reintroducing either S326, S329, or T331 into the ΔABC backbone or mutating specific individual residues within WT FPR2. In contrast to the AC mutant (S326, S329, and T331), restoration of individual phosphorylation sites was unable to reinstate recruitment of either β-arrestin 1 or β-arrestin 2 to the receptor (Fig. 1*D* and *E*, Table 1). However, point mutation of any one of these single amino acids to alanine within the B cluster motif in WT FPR2 significantly reduced both β-arrestin 1 or β-arrestin 2 recruitment to the receptor (Fig. 1*F* and *G*, Table 2).

**Table 1:** Quantification of β-arrestin recruitment performed in Figure 1*B*–*E*

Receptor	Emax ± SD		*p* values	
	β-Arrestin 1	β-Arrestin 2	β-Arrestin 1	β-Arrestin 2
FPR2	0.07818 ± 0.0089	0.0715 ± 0.0087		
ΔABC	0.00138 ± 0.0014	0.01456 ± 0.0029	<0.0001	<0.0001
ΔAB	0.03588 ± 0.0033	0.03962 ± 0.0078	<0.0001	<0.0001
ΔAC	0.06382 ± 0.0065	0.0691 ± 0.0037	0.0102	0.9440 (ns)
ΔBC	0.0009552 ± 0.0033	0.0015 ± 0.0034	<0.0001	<0.0001
ABC + S326A	0.002423 ± 0.0030	0.02168 ± 0.0019	<0.0001	<0.0001
ABC + S328A	−0.001618 ± 0.0009	0.01111 ± 0.0029	<0.0001	<0.0001
ABC + T331A	−0.0008834 ± 0.0012	0.01941 ± 0.0029	<0.0001	<0.0001

ns, not significant.

Emax values were determined by nonlinear regression analysis. Data are expressed as the mean ± SD of at least three independent experiments performed in triplicate and analyzed by one-way ANOVA followed by Dunnett’s multiple comparisons test where *p* values ≤0.05 were significant.

**Table 2:** Quantification of β-arrestin recruitment performed in Figure 1*F* and *G*

Receptor	Emax ± SD		*p* values	
	β-Arrestin 1	β-Arrestin 2	β-Arrestin 1	β-Arrestin 2
FPR2	0.08206 ± 0.0071	0.08544 ± 0.0061		
S326A	0.06227 ± 0.0043	0.06051 ± 0.0042	0.0045	0.0117
S328A	0.04812 ± 0.0044	0.06078 ± 0.0052	0.0001	0.0124
T331A	0.06669 ± 0.0048	0.06112 ± 0.0057	0.0183	0.0168

Emax values were determined by nonlinear regression analysis. Data are expressed as the mean ± SD of at least three independent experiments performed in triplicate and analyzed by one-way ANOVA followed by Dunnett’s multiple comparisons test where *p* values ≤0.05 were significant.

Following activation by WKYMVm, FPR2 exhibits robust colocalization with β-arrestins in enlarged endosomes (22). Characterization of these endosomes revealed them to be positive for transferrin (Tfn), Rab 4, and also Rab 11 but distinct from the described perinuclear recycling compartment (Supp. Fig. S2, *A–C*). Further, these enlarged structures show no Golgi marker staining (Supp. Fig. S2). Combined, these observations are consistent with these structures being derived from the plasma membrane and part of the recycling pathway as previously proposed (22).

**Figure 2:**
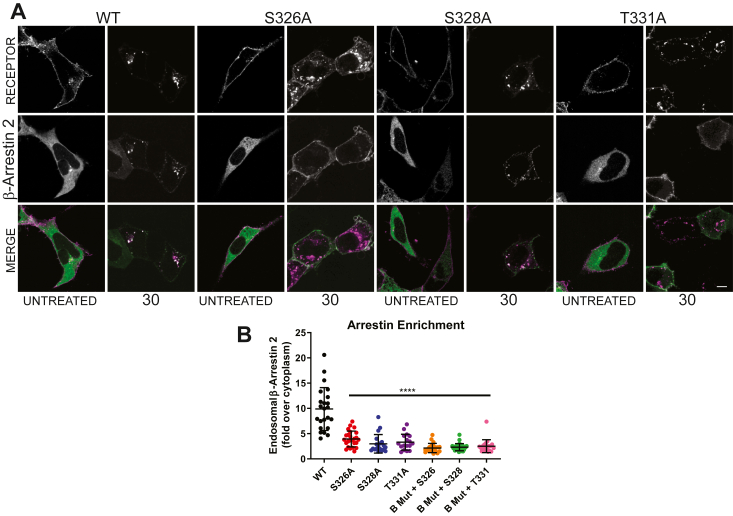
**β-arrestin 2 distribution following activation of FPR2 and putative phospho-site point mutations.** HEK293 cells were cotransfected with N-terminal FLAG-tagged receptor constructs and GFP-β-arrestin 2. Cells were preincubated with FLAG-M1-AlexaFluor 594 followed by 30 min WKYMVm (1 μM) and (*A*) visualized using confocal microscopy, representative images are shown. Scale bars represent 10 μm. *B,* quantification of endosomal enrichment of β-arrestin 2 in cells transiently expressing the indicated receptor and stimulated for 30 min with agonist (17–24 cells from three separate transfections analyzed by the mean of each endosome). Data are expressed as the mean ± SD of at least three independent experiments performed in triplicate and analyzed by one-way ANOVA followed by Dunnett’s multiple comparisons test where ∗∗∗∗*p* ≤ 0.0001. FPR2, formyl-peptide receptor 2; HEK293, human embryonic kidney 293 cell line.

Our previous work has shown that alanine replacement of the whole B cluster in FPR2 impacted endosomal colocalization with β-arrestins (22). Here, we show alanine replacement of any one site in the “B” cluster (S326A, S328A, and T331A), while not preventing internalization of the receptor, was sufficient to reduce the endosomal localization of β-arrestin 2 (Fig. 2*A* and *B*), and the reintroduction of either site in to ΔB backbone (B Mut in Fig. 2*B*) was insufficient to return the endosomal localization of β-arrestin 2. Taken together, these data clearly demonstrate that serine–threonine residues specifically within the B cluster of the FPR2 C-terminus are critical sites for stable association with β-arrestins, most likely because of being substrates for phosphorylation.

### β-arrestin recruitment to FPR2 is predominantly controlled by GRK5

**Figure 3:**
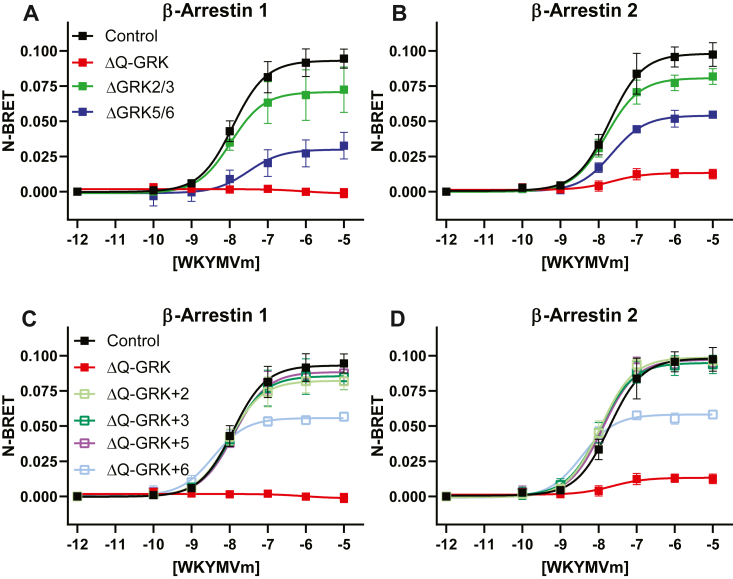
**GRK dependence of β-arrestin recruitment to FPR2.** CRISPR–Cas9-edited cell lines to remove GRKs were transiently transfected with FPR2-RLuc8 and either β-arrestin-1 (*A* and *C*) or β-arrestin-2 (*B* and *D*) tagged with Venus fluorescent protein. *C* and *D,* ΔQ-GRK cells were cotransfected with the indicated replacement GRK. Cells were treated with increasing concentrations of WKYMVm for 10 min before measurement. Data are expressed as the normalized luminescence of acceptor:donor and the mean ± SD of at least three independent experiments performed in triplicate. FPR2, formyl-peptide receptor 2; GRK, G-protein–coupled receptor kinase.

Next, having identified the residues important for β-arrestin recruitment, we investigated which GRKs were important in this process. Using CRISPR–Cas9-edited human embryonic kidney 293 (HEK293) cells (23), deletion of GRKs 2, 3, 5, and 6 (ΔQ-GRK) attenuated the recruitment of both β-arrestin 1 and β-arrestin 2 to FPR2 following agonist stimulation (Fig. 3*A* and *B*, Table 3). Deletion of GRK2/3 caused a small but significant reduction in β-arrestin recruitment to the receptor following agonist exposure. In contrast, deletion of GRK5/6 resulted in an ∼40 to 50% decrease in recruitment. To further investigate these findings, ΔQ-GRK cells were individually transfected with GRKs 2, 3, 5 or 6 and these experiments were repeated. β-arrestin recruitment was completely rescued when either GRKs 2, 3 or 5 were transiently expressed in ΔQ-GRK cells; however, GRK 6 expression only partially recovered this response despite having similar amount of expression (Fig. 3*C* and *D*, Table 3, Supp. Fig S3*F*). However, we did note overexpression of GRK6 elevated baseline measurement indicative of constitutive phosphorylation (23) although this increase was insufficient to explain the reduction in Emax (data not shown). A similar result was observed for FPR1-driven β-arrestin recruitment to the receptor (Suppl. Fig. S3, *A–E*). Taken together, these data suggest that, upon overexpression, any GRK is sufficient to drive β-arrestin recruitment to both FPR1 and FPR2. Under endogenous expression, however, GRKs 6 and, in particular 5, mediate the β-arrestin recruitment following activation by WKYMVm.

**Table 3:** Quantification of β-arrestin recruitment performed in Figure 3

Receptor	Emax ± SD		*p* values	
FPR2	β-Arrestin 1	β-Arrestin 2	β-Arrestin 1	β-Arrestin 2
Control	0.09309 ± 0.0042	0.09803 ± 0.0047		
ΔQ-GRK	−0.001333 ± 0.0027	0.01330 ± 0.0020	<0.0001	<0.0001
ΔGRK2/3	0.07081 ± 0.0064	0.08068 ± 0.0031	<0.0001	<0.0001
ΔGRK5/6	0.02991 ± 0.0051	0.05405 ± 0.0020	<0.0001	<0.0001
ΔQ-GRK +2	0.08218 ± 0.0035	0.09874 ± 0.0029	0.0135	0.9996 (ns)
ΔQ-GRK +3	0.08551 ± 0.0045	0.09488 ± 0.0035	0.1206 (ns)	0.6818 (ns)
ΔQ-GRK +5	0.08843 ± 0.0031	0.09711 ± 0.0034	0.5432 (ns)	0.9994 (ns)
ΔQ-GRK +6	0.05574 ± 0.0021	0.05828 ± 0.0020	<0.0001	<0.0001

ns, not significant.

Emax values were determined by nonlinear regression analysis. Data are expressed as the mean ± SD of at least three independent experiments performed in triplicate and analyzed by one-way ANOVA followed by Dunnett’s multiple comparisons test where *p* values ≤0.05 were significant.

### Internalization of FPR2 is facilitated by GRK expression but does not require β-arrestin recruitment

**Figure 4:**
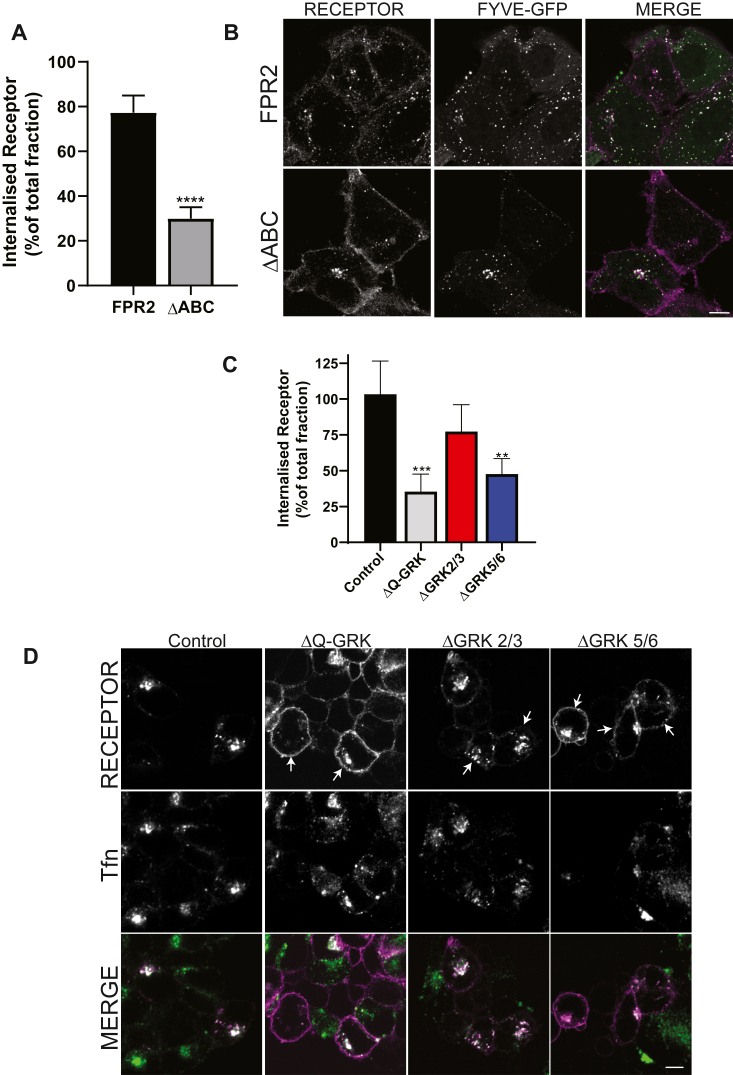
**FPR2 internalization is dependent on C-tail phosphorylation by GRKs.** HEK293 cells were transfected with either N-terminal FLAG-tagged FPR2 or ΔABC (*A*) or in combination with FYVE-GFP (*B*). *A,* cells were preincubated with AlexaFluor-M1-647 and stimulated with 1 μM WKYMVm for 30 min and analyzed using flow cytometry. Internalized receptor was expressed as fold over total surface receptor. *B,* cells were preincubated with AlexaFluor-M1-563 stimulated with 1 μM WKYMVm for 30 min and analyzed by confocal microscopy. *C* and *D,* CRISPR–Cas9-edited cell lines were transiently transfected with FPR2, serum starved for 2 h followed by preincubation with transferrin (Tfn) and AlexaFluor-M1-647 with internalization and microscopy performed as in *A* and *B*. *A* and *C,* data are presented as the mean ± SD of at least three independent experiments performed in duplicate and analyzed using (*A*) unpaired two-tailed *t* tests where ∗∗*p* ≤ 0.001 compared with control or (*C*) one-way ANOVA followed by Dunnett’s multiple comparisons test where ∗∗*p* ≤ 0.01 and ∗∗∗*p* ≤ 0.001 compared with control. *B* and *D,* representative confocal images are shown (receptor in *magenta*, (*B*) FYVE and (*D*) Tfn in *green*, colocalization in *white*). Scale bars represent 10 μm. FPR2, formyl-peptide receptor 2; GRK, G-protein–coupled receptor kinase; HEK293, human embryonic kidney 293 cell line.

Given FPR2 activation was followed by GRK5-dependent β-arrestin recruitment, we next determined the impact of this phenomenon on endocytosis. We found that mutation of the putative phosphorylation sites within the C-tail (ΔABC) resulted in >50% decrease in receptor internalization (Fig. 4*A*). However, although ΔABC remained predominantly on the surface following agonist exposure, internalization was not completely prevented as evidenced by colocalization with an early endosomal marker (GFP-2xFYVE) (Fig. 4*B*). Next, we tested the impact of GRK deletion using our CRISPR–Cas9-edited HEK293 cells. Similar to our previous findings, deletion of all GRK isoforms (ΔQ-GRK) greatly reduced internalization to a level similar to that of the ΔABC mutant (Fig. 4*C*). The same was observed in the absence of GRKs 5/6, but deletion of GRK 2/3 only had a subtle effect. Given the nature of GRKs, these data indicate that C-terminal phosphorylation of FPR2 is required for internalization, and as with β-arrestin recruitment, there is a preference for GRK5/6 for this process. It is worth noting that, as seen with the ΔABC mutant, internalization was not completely abolished with a pool of receptors detectable inside the cell following agonist treatment (compare untreated cells, Supp. Fig S4*D* with Fig. 4*D*). However, surface levels of FPR2 remained notably higher in ΔQ-GRK and ΔGRK 5/6 compared with either ΔGRK 2/3 and particularly control, with Tfn uptake unaffected throughout (as indicated by *arrows*, Fig. 4*D*). Hence, although GRK5/6 are important for mediating FPR2 internalization, there is a pool of receptors that either internalize *via* another GRK isoform (possibly GRK4, which is also expressed in these cells, Supp Fig. S4*C*) or by a GRK-independent mechanism.

**Figure 5:**
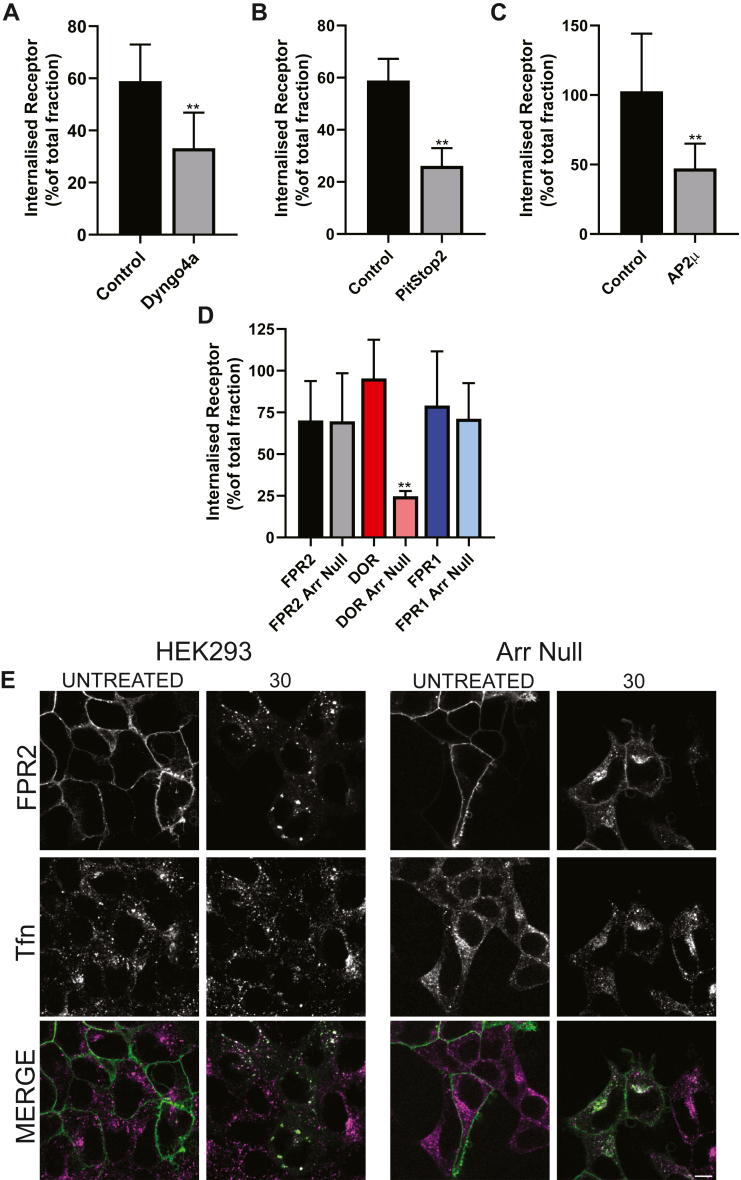
**β-arrestin recruitment is not essential for FPR2 endocytosis.** HEK293 cells stably expressing N-terminal FLAG-tagged FPR2 were pretreated with AlexaFluor-M1-647 and either (*A*) Dyngo4a (40 μM), (*B*) Pitstop2 (20 μM), or (*C*) RNAi against AP2μ, stimulated with 1 μM WKYMVm for 30 min, and analyzed using flow cytometry. Internalized receptor was expressed as percentage of total surface receptor. *D* and *E,* HEK293 cells or CRISPR–Cas9-edited cell lines to remove β-arrestins 1 and 2 (Arr Null) were transfected with N-terminal FLAG-tagged FPR1, FPR2, or DOR. Cells were preincubated with Alexa-M1-647 and stimulated with agonist (1 μM WKYMVm, FPR1/2, 1 μM DADLE, and DOR) for 30 min and analyzed using flow cytometry. Internalized receptor was expressed as percentage of total surface receptor. *E,* HEK293 or Arr Null cells expressing FPR2 were serum starved for 1 h and labeled with AlexaFluor-M1-563 and transferrin (Tfn), stimulated with 1 μM WKYMVm for 30 min, and analyzed by confocal microscopy. Representative confocal images are shown (receptor in *green*, Tfn in *magenta*, and colocalization in *white*). Scale bars represent 10 μm. Data are presented as the mean ± SD of at least three independent experiments performed in duplicate and analyzed using unpaired two-tailed *t* tests where ∗∗*p* ≤ 0.001 compared with (*A*) control or (*B*) DOR. DADLE, [D-Ala-2, D-Leu-5]-enkephalin; DOR, delta opioid receptor; FPR1/2, formyl-peptide receptor 1/2; HEK293, human embryonic kidney 293 cell line.

Next, we determined the importance of CCPs and used a combination of chemical inhibitors and RNAi of different components of this process. In agreement with previous findings, inhibition of dynamin (Fig. 5*A*), clathrin assembly (Fig. 5*B*), or deletion of the AP2 complex (Fig. 5*C*) all reduced the extent of FPR2 internalization, indicating that FPR2 endocytosis was indeed mediated *via* CCPs.

We utilized previously described CRISPR–Cas9-edited cells that do not express either β-arrestin 1 or 2 (Arrestin Null) (24). Having found no difference on cell surface expression of receptors between these cells and WT HEK293s (Fig. S5*D*), we next investigated the impact on FPR2 internalization. As a positive control for our assay, we utilized the delta opioid receptor (DOR) that has a well-characterized internalization profile dependent on β-arrestins (25, 26). As expected, deletion of β-arrestin proteins negatively impacted internalization of DOR in response to [D-Ala-2, D-Leu-5]-enkephalin (Fig. 5*D*, Fig. S5*G*). In contrast, FPR2 internalization was not inhibited by deletion of β-arrestin proteins, similar to previous studies investigating FPR1 (19, 20). Despite FPR2 internalization continuing in the absence of β-arrestins, this process was not *via* a completely distinct pathway, as internalization in Arrestin Null cells was still sensitive to disruption of the AP2 complex, suggesting that FPR2 enters CCPs *via* additional adaptor proteins (Fig. S5*E*). Although deletion of β-arrestins had no effect of FPR2 internalization, there were apparent differences in the pattern of colocalization between β-arrestin and FPR2 (Fig. 5*E*). In control HEK cells, FPR2 exhibited localization in enlarged endosomes, whereas deletion of β-arrestins resulted in a more diffuse pattern throughout the cytoplasm reminiscent of our previous findings when serine and threonine residues were removed from the “B cluster” in the C-tail (22).

**Figure 6:**
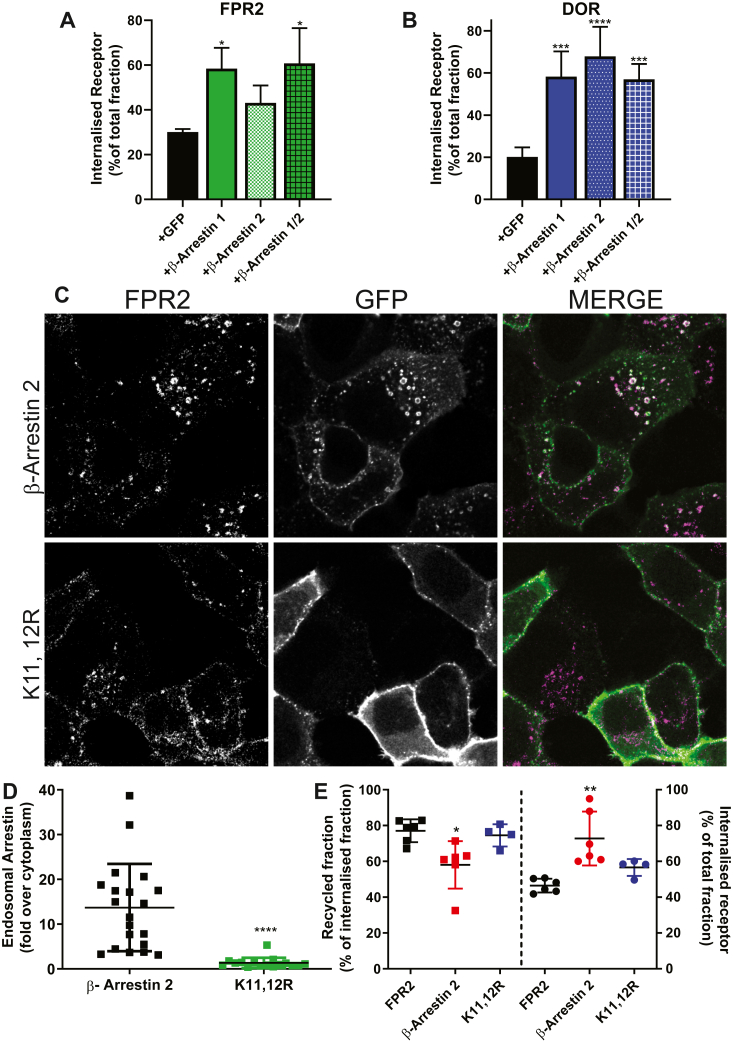
**β-arrestin is important in controlling post endocytic sorting.** Arrestin Null cells transiently expressing N-terminal FLAG-tagged FPR2 (*A*) or DOR (*B*) were cotransfected with GFP, β-arrestin 1-GFP, β-arrestin 2-GFP, or both, labeled with AlexaFluor-M1-647, stimulated with 1 μM WKYMVm (*A*) or DADLE (*B*) for 30 min, and analyzed using flow cytometry. *C,* confocal microscopy of FPR2 Arrestin Null stables cotransfected with either β-arrestin 2-GFP or β-arrestin 2-K11,12R-GFP, and stimulated for 30 min with agonist. *D,* quantification of endosomal β-arrestin 2 intensities shown in *C* (20 and 16 cells from 3 different experimental days). *E,* internalization and recycling experiments were performed using Arrestin Null cells cotransfected with FPR2 and either GFP, β-arrestin 2-GFP, or β-arrestin 2-K11,12R-GFP. Cells were labeled with AlexaFluor-M1-647, stimulated with 1 μM WKYMVm for 30 min, washed with PBS–EDTA (minus Ca^2+^ and Mg^2+^), and returned to the incubator for 90 min before analysis by flow cytometry. Data are presented as the mean ± SD of at least three independent experiments performed in duplicate and analyzed using (*D*) unpaired two-tailed *t* tests or (*A*, *B*, *E*) one-way ANOVA followed by Dunnett’s multiple comparisons test where ∗*p* ≤ 0.05, ∗∗*p* ≤ 0.01,∗∗∗*p* ≤ 0.001, and ∗∗∗∗*p* ≤ 0.0001 compared with control. Representative confocal images are shown (receptor in *magenta*, GFP in *green*, and colocalization in *white*). Scale bars represent 10 μm. DADLE, [D-Ala-2, D-Leu-5]-enkephalin; DOR, delta opioid receptor; FPR2, formyl-peptide receptor 2.

Despite β-arrestins not being an absolute requirement for FPR2 internalization, reintroducing either or both isoforms greatly enhanced this process as was also evident with DOR (Fig. 6*A* and *B*, Fig. S6, *A* and *B*), suggesting that FPR2–β-arrestin interaction can be utilized for internalization, but additional pathways are also available. Further, the interaction is clearly important for the enlarged early endosomes observed following FPR2 internalization. This observation was reinforced by the use of the K11,12R β-arrestin 2 construct, a mutant that also shows reduced endosomal localization with the class B vasopressin receptor (27). We observed that expression of this mutant had a similar effect on FPR2, reducing endosomal localization of β-arrestin 2 and reducing the apparent size of FPR2-containing endosomes (Fig. 6*C* and *D*). Finally, having previously observed that endosomal association of β-arrestin with FPR2 influenced its recycling (22), we investigated whether β-arrestin was an absolute requirement for this process. FPR2 recycling was unaffected in Arrestin Null cells compared with WT HEK293 cells (Suppl. Fig. S5*F*), and somewhat surprisingly, neither was the FPR1 that has previously been suggested to require β-arrestins for efficient recycling (28). Importantly, the reintroduction of β-arrestin 2 reduced the extent of recycling from the internalized FPR2 pool, but enhanced internalization, effects lost when expressing the K11,12R β-arrestin 2 (Fig. 6*E*), indicating that β-arrestins influence the extent of FPR2 recycling by holding the receptor within the early endosome. Taken together, these data suggest β-arrestins may play a dual role in targeting FPR2 to the correct intracellular compartment and controlling plasma membrane redelivery.

### Generation of a β-arrestin-“biased” FPR2

**Figure 7:**
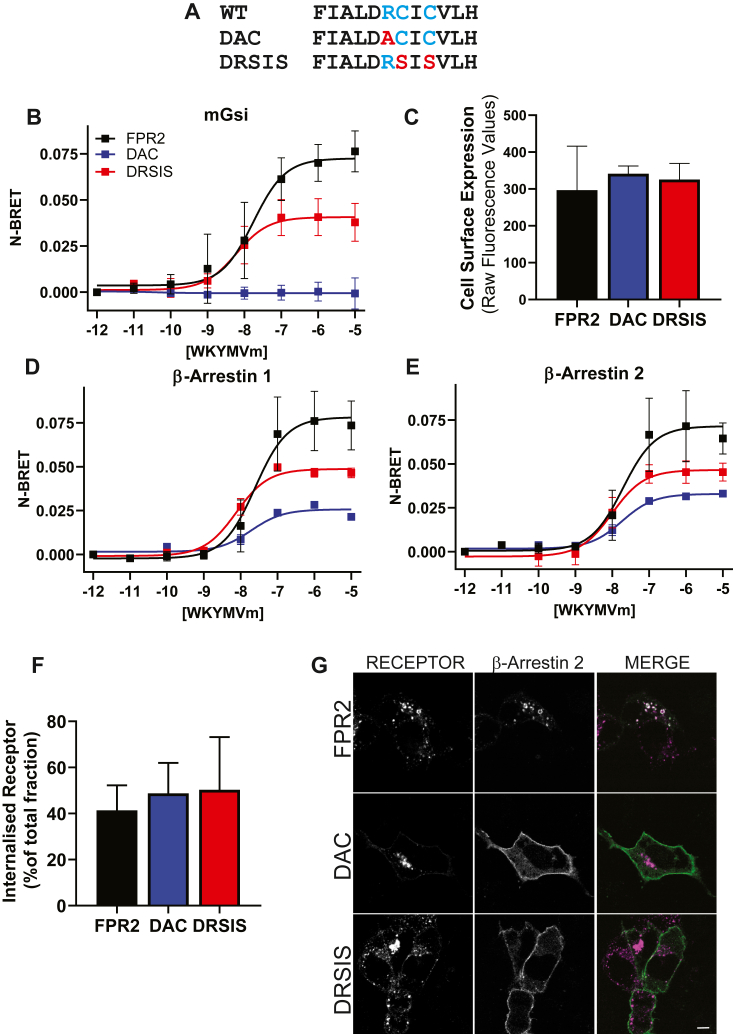
**Generation of a biased FPR2.***A,* schematic of FPR2 mutations investigated. BRET analysis of (*B*) mGsi, (*D*) β-arrestin 1, and (*E*) β-arrestin 2. *B*, *D*, and *E,* HEK293 cells were transfected with N-terminal FLAG-tagged-FPR2 or FPR2 mutants fused C-terminally to RLuc8 along with β-arrestin 1 or 2 fused to Venus. Cells were treated with increasing concentrations of WKYMVm for 10 min before measurement. *C* and *F,* HEK293 cells transiently transfected with N-terminal FLAG-tagged FPR2, or mutants were labeled with AlexaFluor-M1-647, and either (*C*) untreated or (*F*) stimulated with 1 μM WKYMVm for 30 min and analyzed using flow cytometry. *G,* HEK293 cells were cotransfected with FLAG-tagged receptor constructs and GFP-β-arrestin 2. Cells were preincubated with FLAG-M1-AlexaFluor 594 followed by 30 min WKYMVm (1 μM) and visualized using confocal microscopy, representative images are shown (receptor in *magenta*, β-arrestin 2 in *green*, and colocalization in *white*). Data are expressed as the normalized luminescence of acceptor:donor and the mean ± SD of at least three independent experiments performed in triplicate. Scale bars represent 10 μm. BRET, bioluminescence resonance energy transfer; FPR2, formyl-peptide receptor 2; HEK293, human embryonic kidney 293 cell line.

In recent years, a direct role for β-arrestin 2 in G-protein-independent signaling has been proposed for many GPCRs (29, 30). Since β-arrestins play an important role in regulating FPR2 subcellular localization following endocytosis, we investigated whether it was necessary for signaling. Many studies have implicated the DRY motif in the third transmembrane domain as important for G-protein coupling for many GPCRs (31, 32) including for the related FPR1 (DRC motif) (33). Based on these previous studies, we targeted the arginine (R) and cysteine (C) residues within the FPR2 DRC region to generate two FPR2-G-protein coupling mutants, DAC and DRSIS (Fig. 7*A*). We first observed that signaling downstream of G-protein activation was required for neither WKYMVm-dependent FPR2 internalization nor β-arrestin recruitment since, pretreatment with Pertussis toxin (PTx), which inhibits the G_αi_ subunit, had no effect (Suppl. Fig. S7, *A–C*). Mutation of the DRC sequence to DAC abolished G-protein coupling, as measured by recruitment of mGsi, whereas the DRCIC to DRSIS resulted in approximately 50% reduction in Emax when compared with WT FPR2 (Fig. 7*B*, Suppl. Fig. S7*D*). These data could not be attributed to cell surface receptor expression as there was no significant difference in plasma membrane fluorescence as assessed by flow cytometry (Fig. 7*C*). In contrast, although G-protein recruitment was attenuated in the DAC mutant, recruitment of either β-arrestin 1 or 2 was still evident with the latter displaying similar Emax to DRSIS (Fig. 7*D* and *E*, Suppl. Fig S7*D*). However, similar to our observed mGsi recruitment, DRSIS exhibited a 50% reduction in recruitment of both β-arrestin 1 or 2 (Fig. 7*D* and *E*, Fig. S7*D*). Interestingly, although G-protein coupling was either abolished (DAC) or significantly reduced (DRSIS) and despite an evident reduction in β-arrestin recruitment, there was no change in ligand-induced FPR2 endocytosis consistent with the previous data (Fig. 7*F*). Clear internalization of WT and mutant receptors was also observed by confocal microscopy; however, limited endosomal localization with β-arrestin 2 was observed for either DAC or DRSIS following agonist exposure, with β-arrestin 2 remaining enriched at the plasma membrane (Fig. 7*G*, Fig. S7, *E–H*, Fig S6*D*, *upper panels*). It is worth noting, these FPR2 “bias” mutants still contain intact C-terminal serine and threonine putative phosphorylation sites indicating that the endosomal enrichment of β-arrestin requires additional sites outside the C terminus and likely reflect an additional requirement for stable interaction with the receptor core (34).

**Figure 8:**
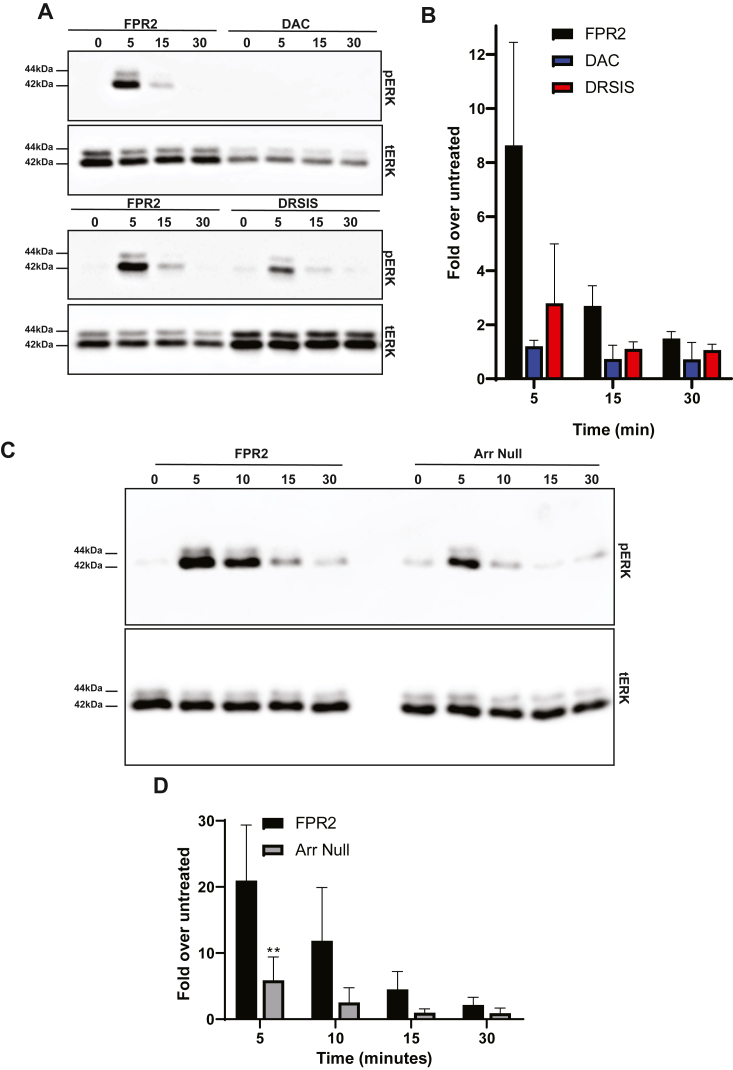
**Deletion of β-arrestin 1 and 2 negatively impacts signal transduction.***A,* HEK293 cells stably expressing N-terminal FLAG-tagged FPR2, DAC, or DRSIS were serum starved for 1 h before stimulation with 1 μM WKYMVm for 0, 5, 15, and 30 min. Cells were lysed, separated by SDS-PAGE, probed for phospho-ERK 1/2 (pERK), and stripped and reprobed for total ERK 1/2 (tERK). Representative blots are shown. *B,* quantification of data shown in *A* where values were normalized to tERK and expressed as fold over untreated (0 time point in representative Western blots). *C,* HEK293 or Arrestin Null (Arr Null) cells transiently expressing N-terminal FLAG-tagged FPR2 were serum starved for 1 h before stimulation with 1 μM WKYMVm for 0, 5, 15, and 30 min. Cells were lysed, separated by SDS-PAGE, probed for phospho-ERK 1/2 (pERK), and stripped and reprobed for tERK. Representative blots are shown. *D,* quantification of data shown in *C* where pERK values were normalized to tERK and expressed as fold over untreated (0 time point in representative Western blots). *B* and *D,* data are presented as mean ± SD of at least three independent experiments analyzed by two-way ANOVA. *B,* FPR2 *versus* DAC (interaction *p* = 0.0207, time *p* = 0.0402, receptor *p* = 0.008); FPR2 *versus* DRSIS (interaction *p* = 0.0379, time *p* = 0.0145, receptor *p* = 0.0187); DAC *versus* DRSIS all nonsignificant. *D,* FPR2 *versus* Arrestin Null (interaction *p* = 0.197, time *p* = 0.00125, receptor *p* = 0.0025), followed by Sidak’s multiple comparison *t* tests where ∗∗*p* ≤ 0.01 compared with 5 min time point. FPR2, formyl-peptide receptor 2; HEK293, human embryonic kidney 293 cell line.

Next, since we generated an “arrestin-biased” FPR2 (DAC), we investigated the impact on signal transduction. In agreement with previous studies (35, 36), FPR2-induced pERK was completely prevented following pretreatment with PTx (Suppl. Fig. S8, *A and B*) indicating signaling was occurring *via* G_αi_ G-protein coupling. When we tested the DAC mutant, which had no mG_si_ interaction and minimal β-arrestin recruitment, pERK signaling was completely abolished (Fig. 8*A*, *upper panels*, 8B). In contrast, DRSIS was still able to elicit some activation of pERK within 5 min WKYMVm exposure, although to a lesser extent, returning to baseline levels at 30 min (Fig. 8*A*, *lower panels*, 8*B*). These data clearly indicate that G-protein coupling is critical for initiating signaling to the ERK1/2 cascade, and that β-arrestin coupling is not sufficient for this process. We therefore determined whether β-arrestins may play a role in ERK1/2 signaling in any capacity. Despite having minimal impact on FPR2 internalization, either siRNA (Suppl. Fig. S8, *C and D*) or CRISPR–Cas9-mediated deletion of β-arrestins 1 and 2 (Fig. 8*C* and *D*) negatively impacted pERK activation. Taken together, these data indicate that an association with β-arrestins can influence the magnitude of signal transduction by FPR2.

## Discussion

FPR2 is an attractive target for the pharmaceutical industry because of agonist-specific activation of proresolution mechanisms with a compound currently in clinical trials for heart failure (10). However, not all compounds targeting FPR2 have made the leap from preclinical models to clinical trials, with “pharmacological tolerance” occurring with repeated use, reportedly driven by internalization of the receptor (15). It is therefore vital to obtain a complete understanding of the molecular pharmacology processes engaged by FPR2 activation that drive this process for the development of future therapeutics. Here, we show FPR2 internalization is dependent on GRKs, most likely *via* the phosphorylation of specific “B cluster” amino acids (*i.e.*, S326, S328, and T331) in the C terminus. In addition, we provide evidence for the first time β-arrestins, although not an absolute requirement for internalization, modulate FPR2 postendocytic transit through intracellular compartments, which we propose serves to control the speed of recycling and, as such, determine the magnitude of downstream signal transduction.

Our previous research found mutation of serine and threonine residues, widely accepted as potential phosphorylation sites in the C terminus of FPR2, altered the pattern of intracellular β-arrestin 1/2 recruitment with deletion in the “B cluster” resulting in redistribution of the receptor from enlarged endosomes to the Rab 11 positive “recycling compartment” and faster recycling (22). We further investigated this finding and showed the importance of this B cluster in facilitating rapid and robust β-arrestin recruitment despite no changes in G-protein coupling. Phosphorylation of serine–threonine residues of GPCRs is typically expected to be performed by GRKs (23). Here, we demonstrate that FPR2-mediated recruitment of both β-arrestin 1 and 2 is sensitive to GRK5/6 deletion with a selective preference for GRK5, although each of GRKs 2, 3, 5 and 6 are sufficient when overexpressed. In this regard, FPR2 falls into the category of GPCRs that are regulated by GRKs 2, 3, 5, 6, albeit with a greater preference, GRK5, 6, than that reported for PTHR (23). Importantly, we see a similar dataset for FPR1, which had previously been reported to be phosphorylated by GRK2, although β-arrestin recruitment was not assessed (37). It may be that overexpression of GRK2 is sufficient to drive phosphorylation, and our data indicate this is likely the case; however, we also reveal that the other GRKs will suffice in the absence of GRK2. It is worth noting that in this study we used the synthetic peptide WKYMVm, and one cannot rule out the prospect of ligand-specific mediation of GRKs and whether different ligands may have different requirements (38, 39). Indeed, an elegant study by Lupisella *et al.* found differential ligand utilization of GRK2 *versus* GRK5 (greater Emax), where BMS-986235 showed a leftward shift in β-arrestin 1 recruitment following overexpression of GRK5 when compared with GRK2, whereas ACT-389949 showed no difference. Interestingly, GRK3 was unable to promote β-arrestin 1 recruitment with either ligand (15). However, it should be noted that FPR1 exhibited comparable results to FPR2 in response to WKYMVm. It would be useful to determine whether the pattern of GRK sensitivity is repeated for other ligands against FPR1. Critically, these other studies have not determined how the different GRKs influence internalization of the FPR2, whereas we show that both β-arrestin recruitment and internalization requires GRK5/6. With that in mind, it is worth drawing attention to the fact that, unlike GRK2/3, GRK5/6 are not regulated by βγ subunits (40) which might explain why PTx treatment showed no effect on neither internalization nor did the G-protein interacting mutants (*i.e.*, DAC, DRSIS).

Internalization of FPR2 has been proposed to be critical in the development of tolerance to FPR2 ligands (15), but a detailed investigation into the mechanism has so far not been undertaken. Numerous studies have shown the critical role β-arrestin interaction has in the internalization of many GPCRs (see Ref. (41) for review). Indeed, Huet *et al.* (36) found that FPR2 internalization was attenuated in mouse embryonic fibroblasts deleted of both β-arrestin 1 and 2. These previous data contrast with studies investigating FPR1 that found internalization to be independent of clathrin and β-arrestins (17, 28). Here, we found no evidence that β-arrestins are absolutely required for FPR2 (nor FPR1) internalization, despite the fact FPR2 internalizes *via* CCPs by a mechanism that likely requires phosphorylation by GRKs. Importantly, arrestin-independent internalization of FPR2 also requires intact CCPs, suggesting the presence of an additional phosphorylation-sensitive adaptor to target FPR2 to CCPs, in the absence of functional arrestins. Our data do not rule out any role for β-arrestins, as overexpression does enhance the extent of FPR2 internalization, and it will be important for future studies to confirm the requirement for β-arrestins in FPR1 and 2 regulation in native immune cells. However, since trafficking of the DOR was substantially altered in parallel studies, we believe our observations to be a genuine point of difference in FPR regulation.

These data report that, similar to our B cluster mutations, β-arrestin 1/2 deletion or inhibition using mutant constructs qualitatively altered the subcellular localization of FPR2. Notably, the prominent enlarged endosomes observed in WT cells were lacking, and instead, a more diffuse pattern of endosomal localization was seen. This is similar to what was observed with the FPR1 where prevention of the AP2 interaction with β-arrestin 2 resulted in perinuclear accumulation (20). This phenotype led to the suggestion that FPR1 interaction with an AP2–β-arrestin 2 complex facilitated recycling, prevention of which resulted in apoptosis (42). However, we were unable to identify evidence that recycling of neither FPR1 nor FPR2 was affected in the absence of β-arrestins. Interestingly, we did find that overexpression of WT β-arrestin 2 in CRISPR–Cas9-edited cells delayed FPR2 recycling. This is in contrast to expression of the mutant β-arrestin 2-K11,12R, suggesting that the sustained endosomal interaction with FPR2 is important for controlling the speed of transit through the intracellular compartments and delivery to the plasma membrane. In support of this hypothesis is the observation that transient GPCR–β-arrestin association as with the β_2_-adrenergic receptor (class A phenotype) results in rapid recycling, whereas a more stable interaction, for example with the V2 vasopressin receptor (class B phenotype), is delayed (27). However, it is important to note that postendocytic trafficking of FPR2 is primarily controlled by a distal recycling motif, removal of which redirects to the lysosome irrespective of a class B arrestin phenotype (22).

Our data suggest, as was observed for FPR1 (20, 28), that β-arrestins are not an absolute requirement for FPR2 endocytosis, yet strongly associate with the receptor in enlarged structures for prolonged periods and seem to play a modulatory role in recovery to the plasma membrane. This is in contrast to experiments performed in mouse embryonic fibroblasts lacking β-arrestins (36); however, since these were undertaken in murine cells whilst our own experiments are performed in human cells, one cannot rule out species differences. In addition, we have shown β-arrestins are not required for internalization using two complementary assays that exclusively follow the trafficking of the mature FPR2 surface pool. Our data raise the question of the precise role of β-arrestins with regard to FPR2 function? Numerous studies have implicated the recruitment of β-arrestins as an important regulator of downstream signaling (29, 30, 43), although the ubiquity of this is currently debated (44, 45). Many GPCRs exhibiting a “class B”-type endosomal interaction with β-arrestins also show prolonged pERK signaling (27, 46). However, our data does not share these findings where maximal activation was found after 5 min WKYMVm exposure, returning to baseline by 30 min. Further, our data clearly show that pertussis toxin pretreatment completely abolished WKYMVm-mediated FPR2 pERK activation and, combined with our “biased” FPR2 (*i.e.*, DAC), which did not recruit G-protein but weakly recruited β-arrestin, being unable to stimulate pERK, support the conclusion there needs to be functional G-protein coupling in order for signal transduction to occur (47). Of note however, activation of pERK was still evident in the absence of β-arrestins (both CRISPR–Cas9 edited and siRNA transfected) although the magnitude of signal was weaker, giving further credence to the hypothesis that β-arrestins regulate the intensity of signal, most likely by scaffolding the Raf1–MEK–ERK cascade. This phenomenon has been observed for other GPCRs, specifically, removal of β-arrestins blunted downstream signal transduction in cells expressing either the parathyroid hormone 1 receptor (48), the free fatty acid receptor 4 (24), the C5a receptor (49) or the β_1_-adrenergic receptor (50). However, investigation into the prototypical β_2_-adrenergic receptor yielded opposite results displaying enhanced pERK signaling that could potentially be attributed in part to increased surface expression (51) or by the rewiring of cellular signaling cascades to compensate for the deletion of β-arrestins (52). Of course, we cannot rule out further roles for β-arrestins in the regulation of other signaling cascades, and it remains an important area of research as to what role β-arrestins might play in FPR2 mediation of the inflammatory response. Importantly, in primary neutrophils, pretreatment with the β-arrestin–β2-adaptin inhibitor barbadin did not prevent FPR2 endocytosis, yet potentiated reactive oxygen species production, but this was also accompanied by increased surface of FPR2 expression (53), highlighting a role of the arrestin–AP2 interaction in a distinct signaling pathway. Finally, it is important to highlight that although biosensor tools have greatly enhanced our knowledge and understanding regarding receptor regulation, the use of any single assay may not provide a complete picture. Minimal changes in β-arrestin recruitment may have a more dramatic impact on subcellular localization than previously appreciated. More relevantly for FPR2, our data draw attention to the finding preventing the recruitment of β-arrestins to FPR2 does not necessarily result in the prevention of endocytosis. The observation that the clinical efficacy of FPR2 ligands appears to be reduced by reduction in surface receptor levels (15) would suggest the development of bias ligands that activate FPR2 without endocytosis to be beneficial. It is therefore critical that future research investigates this independent outcome in addition to the typical β-arrestin recruitment, which, in recent times, has become synonymous with receptor activation.

In summary, it is becoming increasingly clear FPR2 is a therapeutic target ripe for clinical exploitation, however, as with many GPCRs, agonistic tolerance remains a stumbling block for their development (15). Mechanistically, internalization of FPR2 appears to be the critical process in this phenomenon, and as such, understanding this process is vital for translation of these compounds. These data show that the development of the so-called biased ligands (that do not recruit β-arrestins), as has been proposed for other GPCRs such as opioid receptors (54, 55) may not be as valuable, as we show that FPR2 internalization is primarily regulated by GRK5, and not by β-arrestins, which serve to modulate signal transduction by controlling intracellular transit.

## Experimental procedures

### Drugs and reagents

FLAG M1 antibodies were purchased from Merck. AlexaFluor 594, 563, and 647 antibody labeling kits, LysoTracker Red DND-99, and AlexaFluor 633-conjugated Tfn were purchased from Fisher Scientific. WKYMVm was purchased from Tocris, and [D-Ala-2, D-Leu-5]-enkephalin was purchased from Merck. Coelenterazine H was purchased from Cayman Chemicals. Dyngo 4a and Pitstop2 were purchased from Abcam and RNAi from Qiagen (AllStars Negative Control siRNA, functionally verified siRNA against human AP2M1 Hs_AP2M1_7). Total and pERK 1/2 antibodies were purchased from Cell Signaling Technology. Dulbecco's modified Eagle's medium and heat-inactivated fetal calf serum were purchased from Fisher Scientific. Polyethyleneimine (PEI), molecular weight 40,000, was purchased from Polysciences, Inc; poly-d-lysine from Merck; and Vectashield with 4′,6-diamidino-2-phenylindole from Vector laboratories. All other reagents were purchased from standard suppliers.

### Complementary DNA constructs

N-terminal signal sequence FLAG-tagged human FPR1, FPR2, and DOR were generated previously (22, 56). C-terminal-tagged luciferase FPR1 and FPR2 were generated using hiFi DNA assembly based on RLuc8 (a gift from Nevin Lambert, Augusta University). Site-directed mutagenesis was performed to introduce C-tail mutations of FPR2 and generate the β-arrestin 2 K11,12R-GFP mutant (NEB site-directed mutagenesis) as previously described (57). 2xFYVE-GFP, GFP-Rab4, GFP-Rab5, and GFP-Rab11 were a kind gift from Dr Rey Carabeo (University of Nebraska). EGFP–β-Arr1 and EGFP–βArr2 were kind gifts from Prof Mark von Zastrow (University of California, San Francisco). BRET constructs were kind gifts from Kevin Pfleger (The University of Western Australia). GRK complementary DNAs were as previously described (23).

### Cell culture and transfection

Constructs were either transiently or stably expressed in native HEK293 cells or those CRISPR–Cas9 edited to delete β-arrestin 1 and 2 (51) or GRKs (23) and maintained in Dulbecco's modified Eagle's medium containing 10% fetal calf serum. Transfections were performed using PEI at a ratio of DNA:PEI of 1:4 for all studies. Cells were routinely screened to ensure they were free from mycoplasma.

### Immunocytochemistry

Cells transiently or stably expressing N-terminal FLAG-tagged receptor constructs alone or in combination with EGFP-βArr2 or 2x FYVE-GFP were seeded onto glass coverslips coated in poly-d-lysine and grown to ∼50 to 60% confluency and incubated with AlexaFluor-M1-563 or AlexaFluor-M1-598 (1:1000 dilution) for 30 min prior to experimentation to label cell surface receptors as described previously (22). For Tfn experiments, cells were serum-starved for 2 h and labeled with AlexaFluor-633-conjugated Tfn (Invitrogen) in the 30 min before agonist stimulation (1:200 dilution). Cells were subsequently stimulated with WKYMVm for 30 min, fixed, quenched, and mounted onto coverslips. For colocalization of receptors with β-arrestin 2 at 30 min of WKYMVm treatment, at least 30 cells from three independent experiments were analyzed and quantified using ImageJ (rsb.info.nih.gov/ij/), by drawing a region of interest around the endosome in the red channel (receptor) and measuring the mean intensity of the green channel (β-arrestin 2). This was then expressed as fold increase compared with the green intensity in the cytoplasm (no red staining).

### Flow cytometry

Cells transiently or stably expressing N-terminal FLAG-tagged receptor constructs alone or in combination with, EGFP, EGFP-β-arrestin 2, or mutant were incubated with AlexaFluor-M1-647 for 30 min prior to experimentation as described previously (22, 58). This feeding protocol labels the surface pool of receptors with little to no background (<1% of total binding to transfected cells). To measure internalization, cells were stimulated for 30 min followed by a PBS–EDTA (2 mM) wash. For recycling experiments, following 30 min agonist stimulation to drive internalization, cells were washed and then returned to the incubator for 90 min in the presence of PBS–EDTA. Cells were subsequently pelleted at 1500 rpm and resuspended in PBS (containing Ca^2+^ and Mg^2+^) and analyzed using the Attune NxT.

### BRET analysis of β-arrestin and mini-Gsi recruitment

HEK293 cells or those CRISPR–Cas 9 edited to delete GRKs were grown on 6well plates until 90 to 95% confluency was reached. Initial optimization studies revealed that low ratios of β-arrestin to receptor blunted any responses, and thereafter for all measurements of direct interaction, 400 ng of FPR1 or 2-RLuc8 (donor) was cotransfected with Venus-tagged (acceptor) mGsi, β-arrestin 1, or β-arrestin 2 at a ratio of 4:1 (acceptor:donor). The following day, cells were replated and seeded into white opaque 96-well plates. Cells were assayed 24–48 h post-transfection as described previously (59, 60). Briefly, cells were incubated at 37°C in Hank’s balanced salt solution for 30–40 min followed by 5 μM Coelenterazine H for 10 min. Cells were then stimulated with increasing concentrations of WKYMVm and analyzed using ClarioStar Plus plate reader (BMG Labtech) at 10 min post agonist addition. Luminescence and fluorescence values were measured at 460/30 nm (donor) and 480/40 nm (acceptor), and the ratio of acceptor:donor was determined and normalized by subtraction of the ratio observed in untreated cells.

### Western blotting

HEK293 cells stably expressing FPR2 or mutant constructs or CRISPR–Cas9-edited Arrestin Null cells transiently transfected with FPR2 were seeded onto 12-well plates and grown to confluency and serum starved for 1 h prior to experimentation. For experiments with PTx, cells were incubated overnight (16 h) with 100 ng/ml PTx. Cells were stimulated with WKYMVm for 5, 10, 15, 30, or 60 min; in some cases, 1 μM phorbol-12-myristate-13-acetate was used as a control and lysed immediately in 100 μl of sample buffer containing DTT, heated to 95^o^C, separated using a 10% Tris–glycine SDS-PAGE gel, and transferred to nitrocellulose. Membranes were probed for pERK 1/2 (Thr202–Tyr204), stripped and reprobed for total ERK 1/2, β-tubulin, or vinculin as a loading control (all Cell Signaling Technology), and visualized by enhanced chemiluminescence.

### Statistics

Data were analyzed using unpaired two-tailed *t* tests, one-way or two-way ANOVA followed by multiple comparison *t* tests between corresponding time points where appropriate using GraphPad Prism 6.0 software (GraphPad Software, Inc). Specific details are listed in the respective figure legends.

## Data availability

All data are contained within the article, and images of replicate Western blots and microscopy are available on request from Dr Dawn Thompson (dthompson@abdn.ac.uk).

## Supporting information

This article contains supporting information.

## Conflict of interest

The authors declare that they have no conflicts of interest with the contents of this article.
