# Supplementary material for: GRK5 regulates endocytosis of FPR2 independent of β-arrestins
Source: J Biol Chem. 2024 Dec 18;301(2):108112. doi: 10.1016/j.jbc.2024.108112 (PMC11773488; doi:10.1016/j.jbc.2024.108112)
Supplement: Supplementary Figures [file mmc1.pdf]

Supporting Information

GRK5 regulates endocytosis of FPR2 independent of β-Arrestins.

Christine E Jack[1], Emily M Cope[1], Laura Lemel[2], Meritxell Canals[2], Julia Drube[3], Carsten Hoffmann[3], Asuka Inoue[4,5], James N Hislop[1]*, Dawn Thompson[1]*

[1.] School of Medicine, Medical Sciences and Nutrition, Institute of Medical Sciences, University of Aberdeen.

[2.] Division of Physiology, Pharmacology and Neuroscience, School of Life Sciences, University of Nottingham, Nottingham

[3.] Institut für Molekulare Zellbiologie, CMB—Center for Molecular Biomedicine, Universitätsklinikum Jena; Friedrich-Schiller-Universität Jena, Hans-Knöll-Straße 2, D-07745, Jena, Germany

[4.] Graduate School of Pharmaceutical Sciences, Tohoku University, Sendai, Miyagi, 980-8578, Japan.

[5.] Graduate School of Pharmaceutical Sciences, Kyoto University, Kyoto, 606-8501, Japan

***Address Correspondence to:**

Dr Dawn Thompson
School of Medicine, Medical Sciences and Nutrition
Institute of Medical Sciences
University of Aberdeen
Aberdeen, AB25 2ZD
United Kingdom
Tel: +44 (0)1224 437513
Fax: +44 (0)1224 437411
Email: dthompson@abdn.ac.uk
OR
Dr James Hislop
School of Medicine, Medical Sciences and Nutrition
Institute of Medical Sciences
University of Aberdeen
Aberdeen, AB25 2ZD
United Kingdom
Tel: +44 (0)1224 437399
Fax: +44 (0)1224 437411
Email: james.hislop@abdn.ac.uk

Experimental Procedures

Cell Culture and Transfection: For transient β-Arrestin knockdown studies, siRNA – duplexes (Functionally verified siRNA against human ARRB1 – Hs_ARRB1_11 and human ARRB2 – Hs_ARRB2_10, Qiagen), were transfected at 60 pmol/60mm dish with Lipofectamine RNAiMax (Invitrogen), according to manufacturers' instructions, replated 48 hours post-transfection and then cultured for a further 24 hours. For PTx treatment, cells were incubated overnight (16h) in 100ng/ml PTx prior to experimentation.

RNA extraction and qPCR: Cells were lysed in Trizol reagent (Sigma, UK) and RNA isolated using phenol/chloroform extraction according to manufacturer's instructions. RNA was then synthesized into cDNA using tetrokits (Bioline) and subjected to qPCR analysis using Takyon and LightCycler 480 (Roche). Gene expression of GRK 2-6 determined relative to the reference gene 28S (Supp.Table S1).

Supplemental Table S1

Gene	Forward Primer (5'-3')	Reverse Primer (5'-3')
***ADRBK1* (GRK2)**	ATGCATGGCTACATGTCCA	ATCTCCTCCATGGTCAGCAG
***ADRBK2* (GRK3)**	TGAAAGCCTTCGAGGTGACAT	TCGTCCAATAATCCTATGCACAC
GRK4	CGTCTCTTCAGGCAGTTCTGTG	GACAGTCCACAATCACTTCGGTC
GRK5	ACCTGAGGGGAGAACCATTC	TGGACTCCCCTTTCCTCTTT
GRK6	AAAACACCTTCAGGCAATACCG	AGGCCAAGCTCACTACAAACCTA
**28S**	GCCTAGCAGCCGACTTACAA	AAATCACATCGCGTCAACAC

Supplemental Figure S1

(A) BRET analysis of mGsi recruitment to FPR2 and ∆ABC. HEK293 cells were transfected with N-terminal FLAG-tagged FPR2 or FPR2 mutants fused C-terminally to RLuc8 along with mGsi Venus. Cells were treated with increasing concentrations of WKYMVm for 10 mins before measurement. Emax of (B) G-protein recruitment. Data is expressed as the normalised ratio luminescence of acceptor:donor and the mean ± SD of at least three independent experiments performed in triplicate and EMax analysed by unpaired two tailed student t test.

Supplemental Figure S2: Subcellular localisation of FPR2.

HEK293 cells were transfected with N-terminal FLAG-tagged receptor construct FPR2 alone (A) or co-transfected with (B) GFP-Rab4, (C) GFP-Rab11 or (D) pmTurq2-Golgi. Cells were pre-incubated with FLAG-M1- Alexa Fluor® 594 followed by 30min WKYMVm (1µM) and (A) visualised using confocal microscopy. For Transferrin (Tfn) experiments, cells were serum starved for 2hrs before incubation with Tfn prior to assay. Representative images are shown (receptor in magenta, Tfn, GFP-Rab4, GFP-Rab11 and pmTurq2-Golgi in green, colocalization in white). Scale bars are 10µm.

Supplemental Figure S3: GRK dependence of β-Arrestin recruitment to FPR1

CRISPR/Cas9 edited cell lines to remove GRKs were transiently transfected with N-terminal FLAG-tagged receptor FPR1-RLuc8 and either β-Arrestin 1 (A and C) or β-Arrestin 2 (B and D) tagged with Venus fluorescent protein, (C-D) ΔQ-GRK cells were co-transfected with the indicated replacement GRK. Cells were treated with increasing concentrations of WKYMVm for 10 mins before measurement. (E) Emax of β-Arrestin recruitment. Data is expressed as the normalised ratio luminescence of acceptor:donor and the mean ± SD of at least three independent experiments performed in triplicate and analyzed by one-way ANOVA followed by Dunnett’s multiple comparisons test. (F) Gene expression of GRKs following transfection of indicated cDNA. Data is expressed as ΔCt where lower values indicated higher expression.

Supplemental Figure S4: FPR2 internalization is dependent on C-tail phosphorylation by GRKs.

HEK293 cells or CRISPR/Cas9 edited cell lines were transfected with either N-terminal FLAG-tagged FPR2 or ΔABC (A, B) Cells were pre-incubated with Alexa Fluor® -M1-647 and total surface expression analysed using flow cytometry. Total surface receptor expression was expressed as percentage of control. (C) Gene expression of GRKs 2-6 as analysed by RT-qPCR. Data is expressed as ΔCt where lower values are indicative of increased expression. Note, CRISPR/Cas9 edited cells (Q-GRK) still express mRNA for GRKs. (D) CRISPR/Cas9 edited cell lines were transiently transfected with FPR2, serum starved for 2h followed by pre-incubation with Transferrin (Tfn) and Alexa Fluor® -M1-594. Untreated samples were analysed by microscopy. (A, B) Data is presented as the mean ± SD of at least three independent experiments performed in duplicate and analysed using (A) unpaired two-tailed t tests or (B) one-way ANOVA followed by Dunnett’s multiple comparisons test. (D) Representative confocal images are shown (receptor in magenta, Tfn in green, colocalization in white). Scale bars are 10µm.

Supplemental Figure S5: FPR1 and FPR2 recycle in the absence of β-Arrestin 1 and 2.

HEK293 cells stably expressing N-terminal FLAG-tagged FPR2 were pretreated with Alexa Fluor® -M1-647 and either (A) Dyngo4a (40µM), (B) Pitstop2 (20µM) or (C) RNAi against AP2µ and untreated samples analysed using flow cytometry. Total surface receptor was expressed as percentage of control. (D-F) HEK293 cells or CRISPR/Cas9 edited cell lines to remove β-Arrestins 1 and 2 (Arr Null) were transfected with N-terminal FLAG-tagged FPR1, FPR2 or DOR or AP2µ RNAi and pre-incubated with Alexa-M1-647. Cells were either (D) untreated or stimulated with agonist (1µM WKYMVm) for 30 mins (E, F), washed with PBS-EDTA and incubated for 90min to facilitate receptor recovery (F). Analysis was performed using flow cytometry as in A-C. (D) Total surface receptor was expressed as percentage of control, (E) Internalised receptor was expressed as percentage of total fraction, (F) recycled receptor was expressed as percentage of internalised fraction. (G) HEK293 or Arr Null cells expressing DOR were labelled with Alexa Fluor® -M1-563 and with 1µM DADLE for 30 mins and analysed by confocal microscopy. Representative confocal images are shown. Scale bars are 10µm. Data is presented as the mean ± SD of at least three independent experiments performed in duplicate and analysed using unpaired two-tailed t tests.

Supplemental Figure S6: β-Arrestin is important in controlling post endocytic sorting.

(A-C) Cell surface receptor expression of N-terminal FLAG-tagged FPR2 (A, C) and DOR (B) in Arr Null cells transiently co-expressing (A, B) GFP, β-Arrestin 1-GFP, β-Arrestin 2-GFP or a combination of 1 and 2 or (C) N-terminal FLAG-tagged FPR2 co expressing GFP, β-Arrestin 2-GFP or β-Arrestin 2-K11,12R-GFP. Cells were labelled with Alexa Fluor® -M1-647, analysed using flow cytometry and expressed as percentage of +GFP control. (D) Confocal microscopy of untreated FPR2 Arr Null stables co-transfected with either β-Arrestin 2-GFP or β-Arrestin 2-K11,12R-GFP Data is presented as the mean ± SD of at least three independent experiments performed in duplicate and analysed using (A-C) one-way ANOVA followed by Dunnett's multiple comparisons test. Representative confocal images are shown (Receptor in magenta, GFP in green, colocalization in white). Scale bars are 10µm.

Supplemental Figure S7: Gi mediated signalling is not required for FPR2 internalisation.

(A) HEK293 cells were co-transfected with N-terminal FLAG-tagged receptor constructs and GFP-β-Arrestin 2 and pre-incubated with 100ng/ml PTx for 16 hours. Subsequently cells were labelled with FLAG-M1 conjugated to Alexa Fluor®-594 30 mins prior to experimentation and either untreated or followed by 30min WKYMVm (1µM) and visualised using confocal microscopy, representative images are shown (receptor is in magenta, GFP-β-Arrestin 2 in green, colocalization is in white), scale bar is 10µm (B) Quantification of endosomal

enrichment in (A), 22-26 cells from three separate transfections analysed by the mean of each endosome). (C) Cells stably expressing FPR2 were pre-incubated with 100ng/ml PTx for 16 hours prior to labelling with Alexa Fluor® -M1-647 and stimulated with increasing concentrations of WKYMVm for 30 mins and analysed using flow cytometry. Internalised receptor was expressed as percentage of total fraction. (D) Emax of mGsi and β-Arrestins. (E) HEK293 cells were co-transfected with FLAG-tagged receptor constructs and GFP-β-Arrestin 2 and pre-incubated with FLAG-M1- Alexa Fluor® 594 and visualised using confocal microscopy, representative images are shown (Receptor in Magenta, β-Arrestin 2 in green, colocalization in white). (F-H) Graphs showing normalised intensity of receptor (Red) or β-Arrestin 2 (green) obtained from dotted line in Fig 7G. Data is expressed as the mean ± SD of at least three independent experiments performed in triplicate. (D) Data was analysed by one-way ANOVA followed by Dunnet's post comparisons test.

Supplemental Figure S8: WKYMVm mediated FPR2 signal transduction is inhibited by PTx and blunted in the absence of β-Arrestins. (A) HEK293 cells stably expressing N-terminal FLAG-tagged FPR2 were untreated or incubated overnight with 100ng/nl PTx and serum starved for 1h before stimulation with 1µM WKYMVm for 0, 5, 10, 15, 30 and 60 mins. Cells were lysed, separated by SDS-PAGE, probed for phospho-ERK 1/2 (pERK) and stripped and re-probed for β-Tubulin. Representative blots are shown. (B) Quantification of data shown in A where values were normalised to β-Tubulin and expressed as fold over untreated (0 time point in representative Western blots). (C) HEK293 cells stably expressing N-terminal FLAG-tagged FPR2 were transfected with control or β-Arrestin 1/2 (β-Arr 1/2) siRNA 72 hr before experimentation. Cells were serum starved 1h before stimulation with 1µM WKYMVm for 0, 5, 15 and 30 mins. Cells were lysed, separated by SDS-PAGE, probed for phospho-ERK 1/2 (pERK) and β-Arr 1/2, stripped and reprobed for Vinculin (Vinc) and total ERK (tERK). Representative blots are shown. (D) Quantification of data shown in C where pERK values were normalised to tERK and expressed as fold over untreated (0 time point in representative Western blots). (B, D) Data is presented as mean ± SD of at least 3 independent experiments analyzed by two-way ANOVA, (B) FPR2 Control vs PTx -interaction $p=0.0003$, time $p=0.0003$, receptor $p=0.0018$ (D) Control vs β-Arr 1/2 siRNA -interaction $p=0.518$, time $p=0.0052$, receptor $p=0.0289$,followed by Sidak's multiple comparison t tests where ****$p<0.0001$ (FPR2 Control vs PTx) .

A.

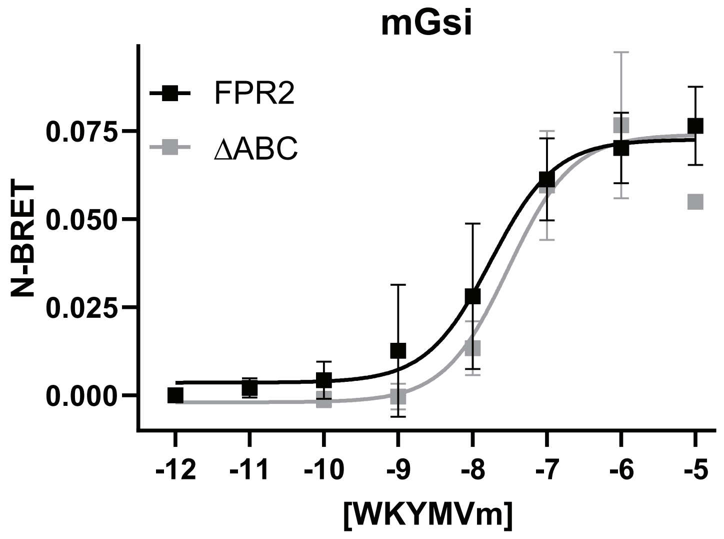

B.

RECEPTOR	mGSi	
	Emax ± SD	p values
FPR2	0.07255 ± 0.0089	
ΔABC	0.07397 ± 0.0100	0.8505 (ns)

Supplemental Figure S1

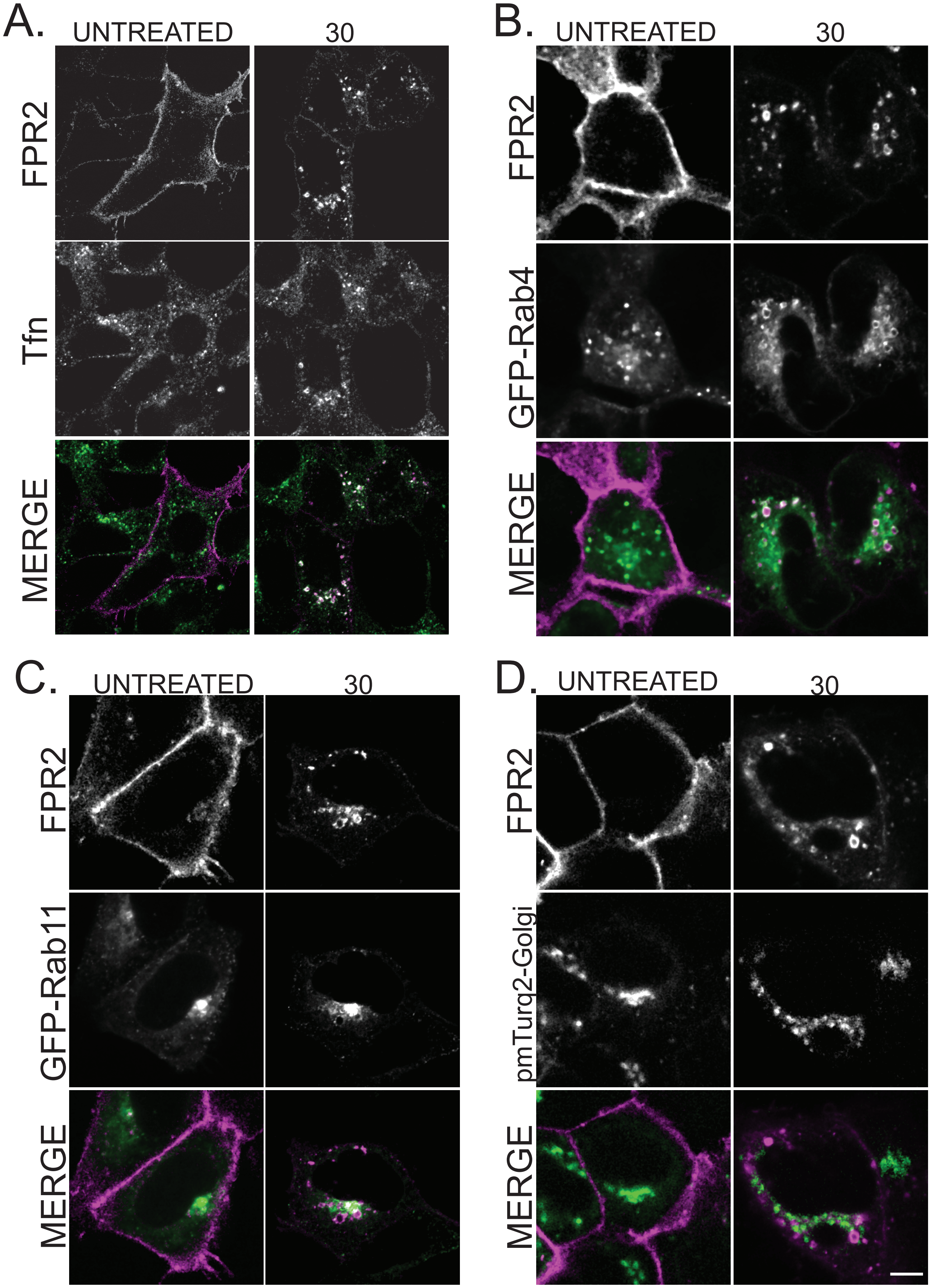

Supplemental Figure S2

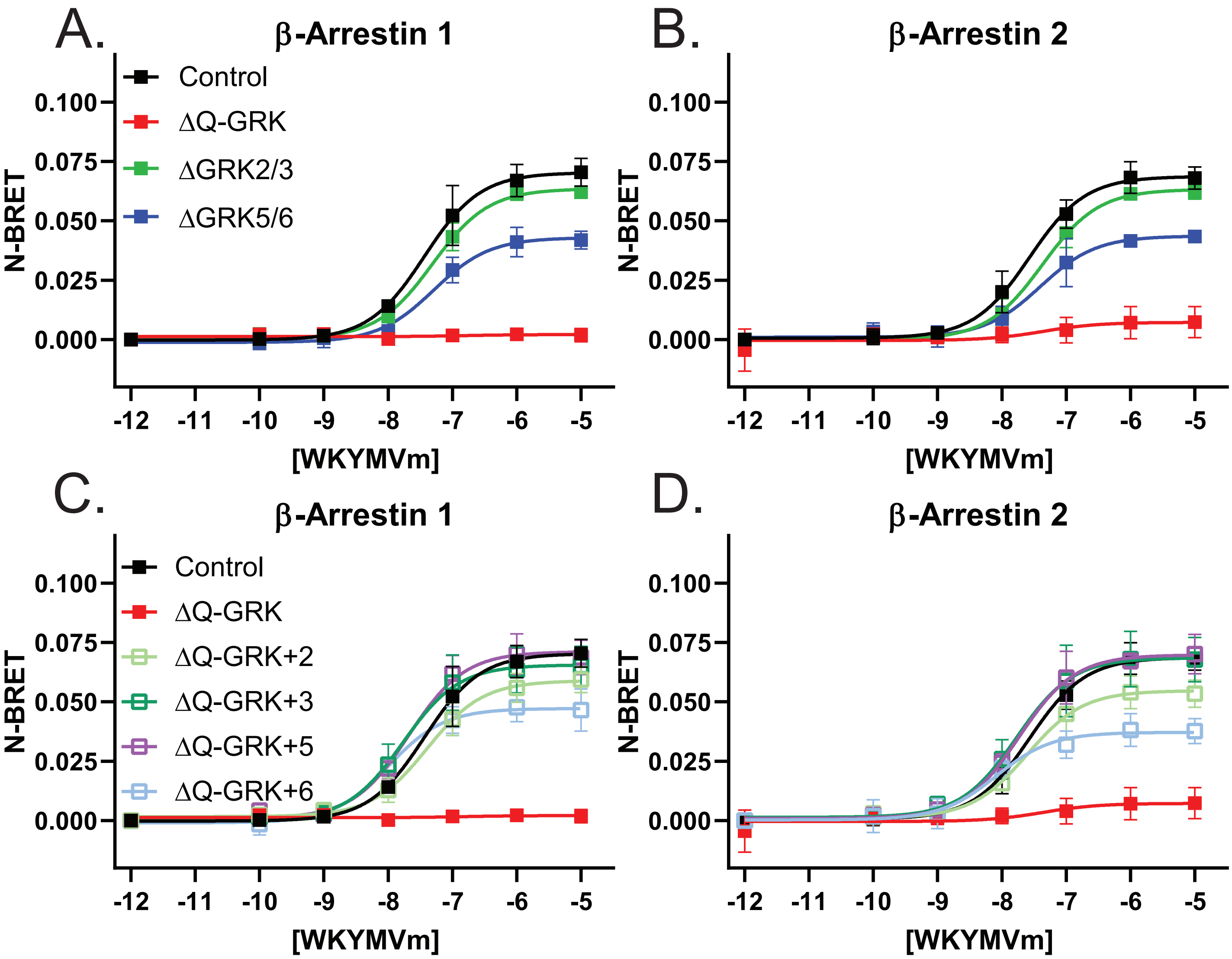

E.

RECEPTOR	Emax ± SD		p values	
**FPR1**	**β-Arrestin 1**	**β-Arrestin 2**	**β-Arrestin 1**	**β-Arrestin 2**
**Control**	0.07031 ± 0.0041	0.06869 ± 0.0035		
**ΔQ-GRK**	0.002107 ± 0.0020	0.007234 ± 0.0043	<0.0001	<0.0001
**ΔGRK2/3**	0.06352 ± 0.0021	0.06328 ± 0.0025	0.0722 (ns)	0.3208 (ns)
**ΔGRK5/6**	0.04291 ± 0.0030	0.04361 ± 0.0034	<0.0001	<0.0001
**ΔQ-GRK +2**	0.05890 ± 0.0031	0.05470 ± 0.0035	0.0014	0.0008
**ΔQ-GRK +3**	0.06555 ± 0.0044	0.06851 ± 0.0052	0.3106 (ns)	>0.9999 (ns)
**ΔQ-GRK +5**	0.07111 ± 0.0037	0.06976 ± 0.0035	0.9996 (ns)	0.9994 (ns)
**ΔQ-GRK +6**	0.04720 ± 0.0028	0.03718 ± 0.0032	<0.0001	<0.0001

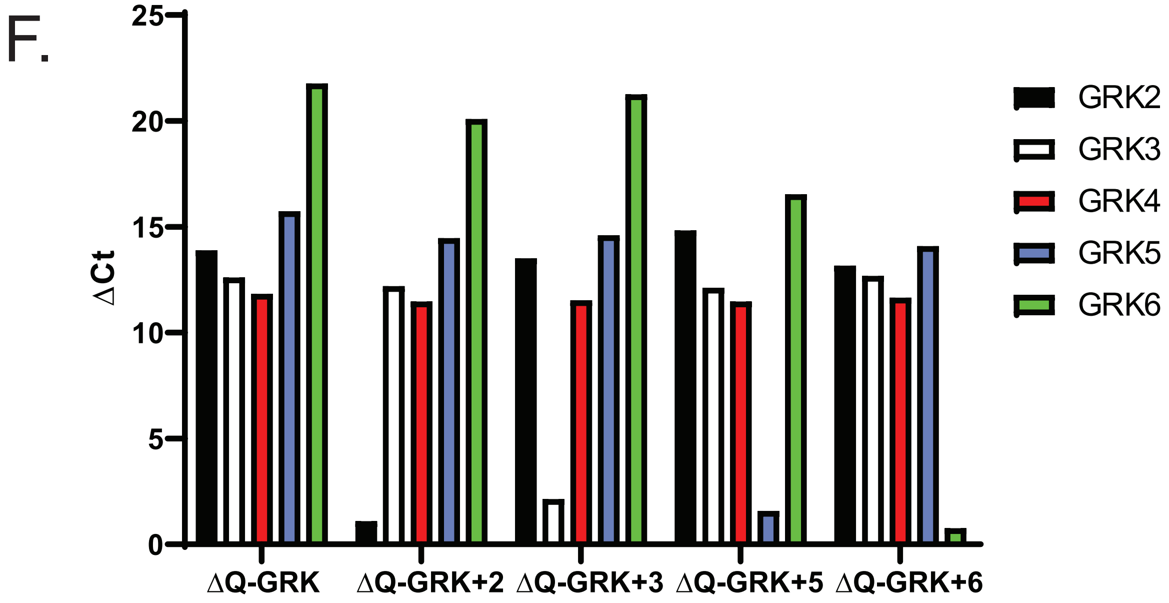

Supplemental Figure S3

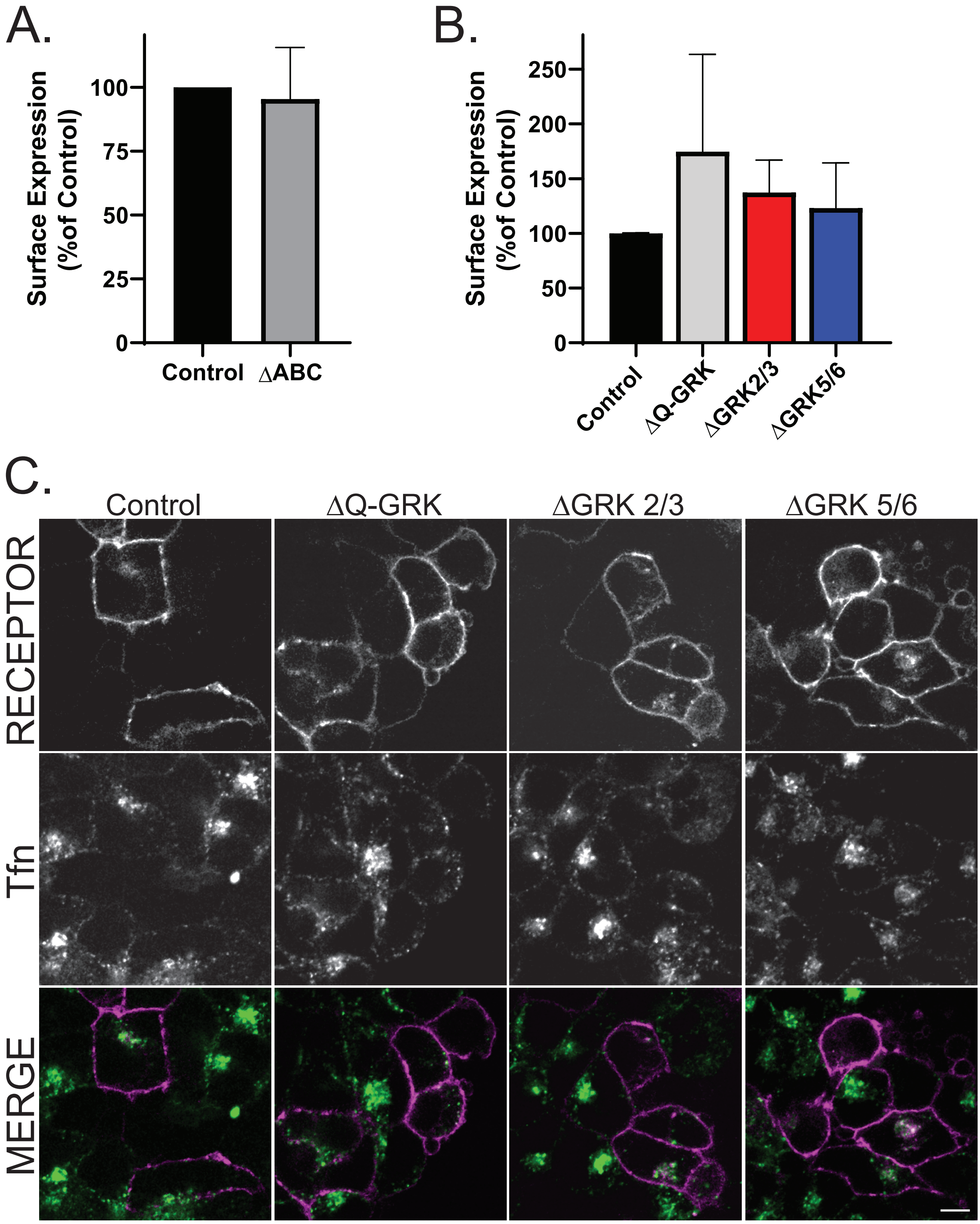

Supplemental Figure S4

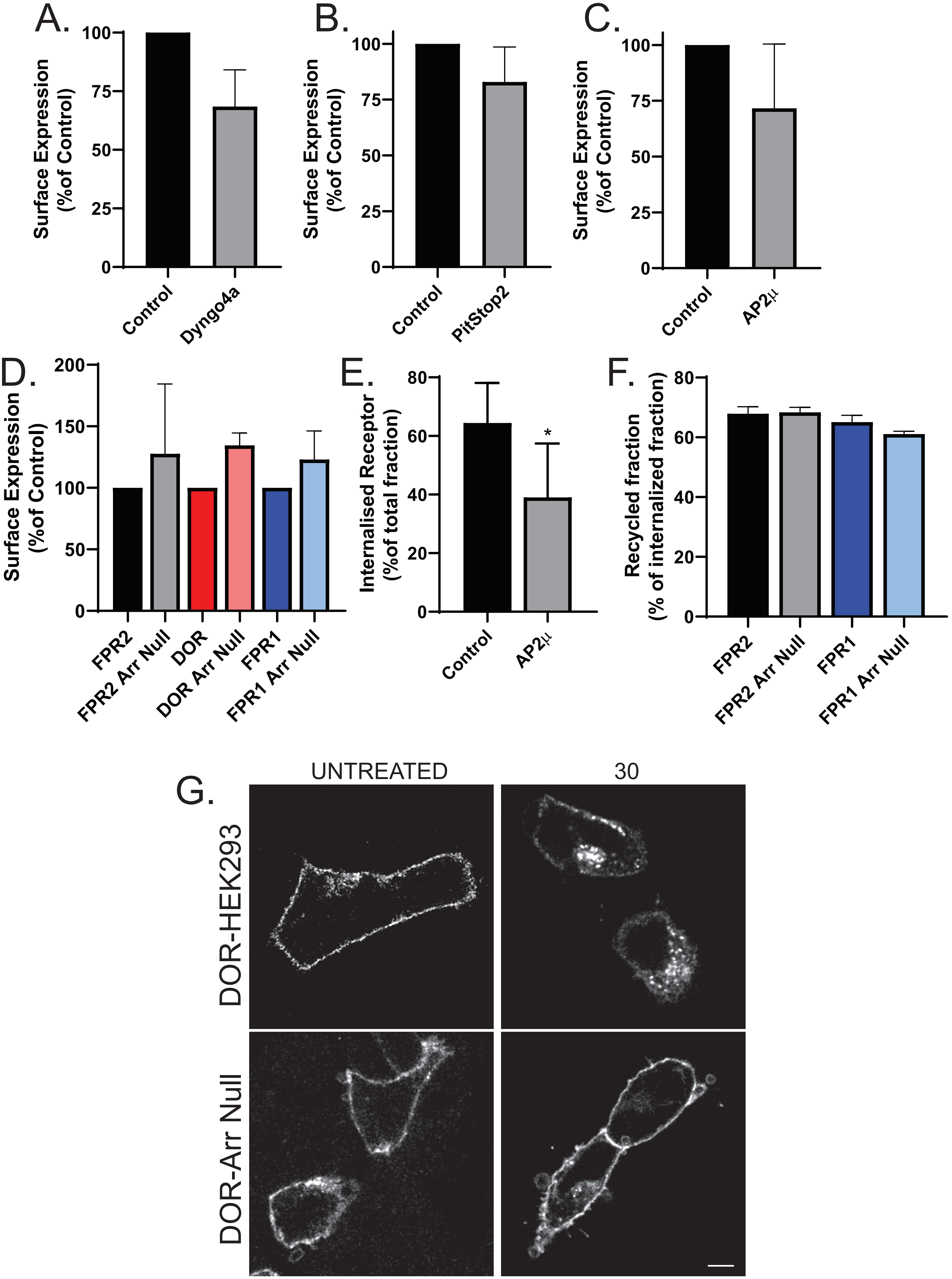

Supplemental Figure S5

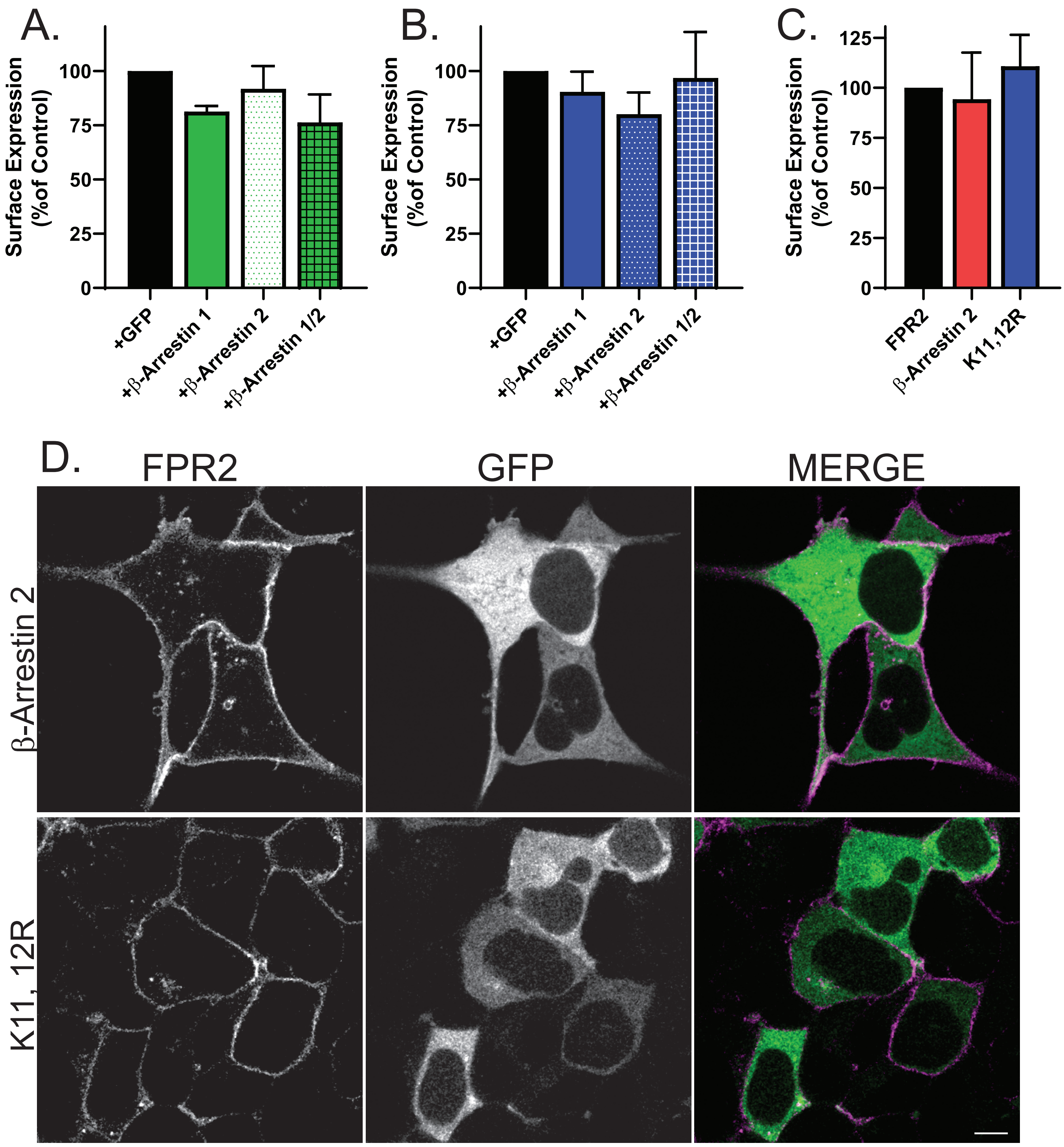

Supplemental Figure S6

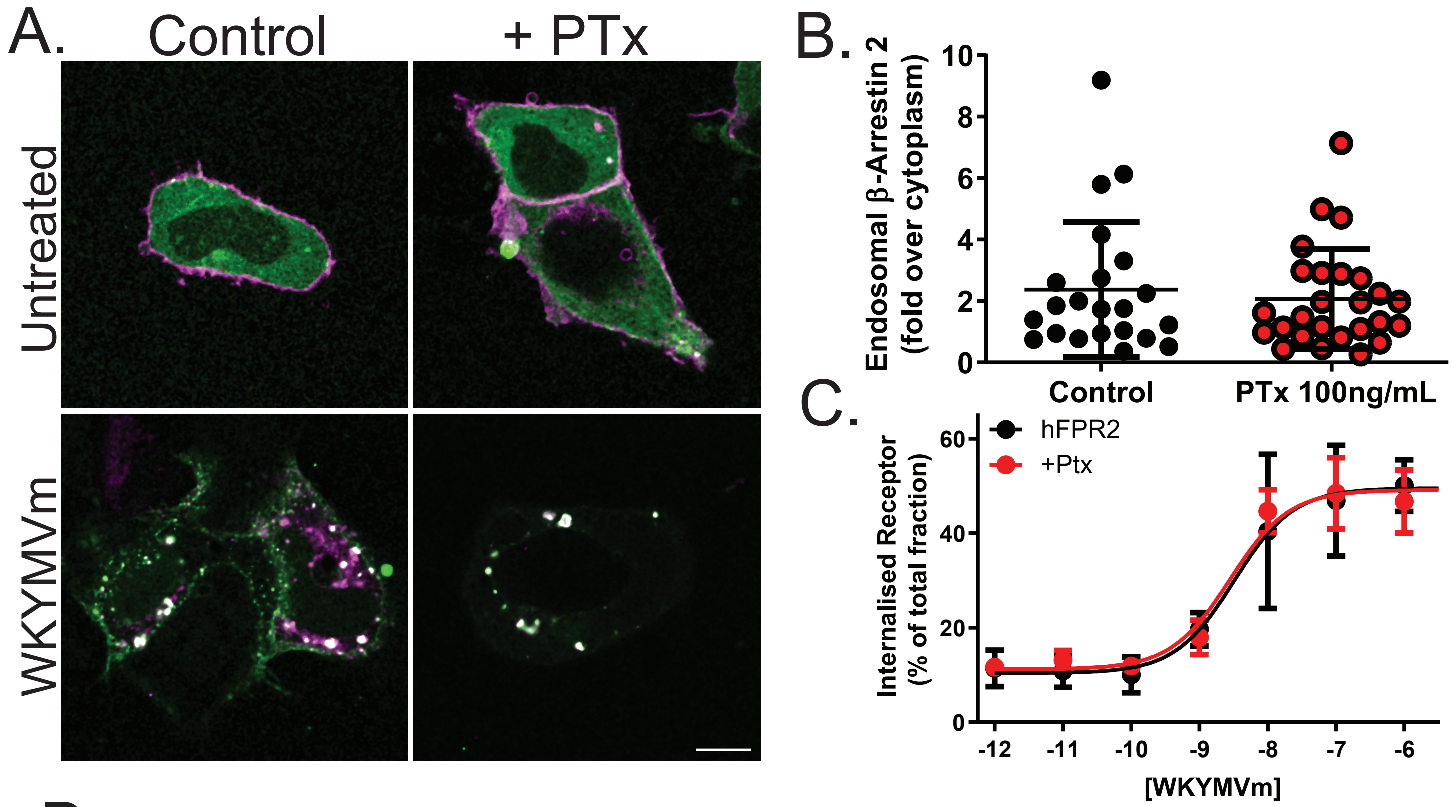

D.

RECEPTOR	Emax ± SD			p values		
	mGsi	β-Arrestin 1	β-Arrestin 2	mGsi	β-Arrestin 1	β-Arrestin 2
**FPR2**	0.07255 ± 0.0089	0.07818 ± 0.0089	0.07150 ± 0.0087			
**DAC**	-0.0005488 ± 0.0016	0.02570 ± 0.0021	0.03292 ± 0.0012	<0.0001	<0.0001	<0.0001
**DRSIS**	0.04073 ± 0.0045	0.04871 ± 0.0021	0.04656 ± 0.0035	* 0.0008<br># 0.0002	*0.0006<br>#0.0060	*0.0019<br>#0.0747<br>(ns)

* compared to FPR2. # compared to DAC

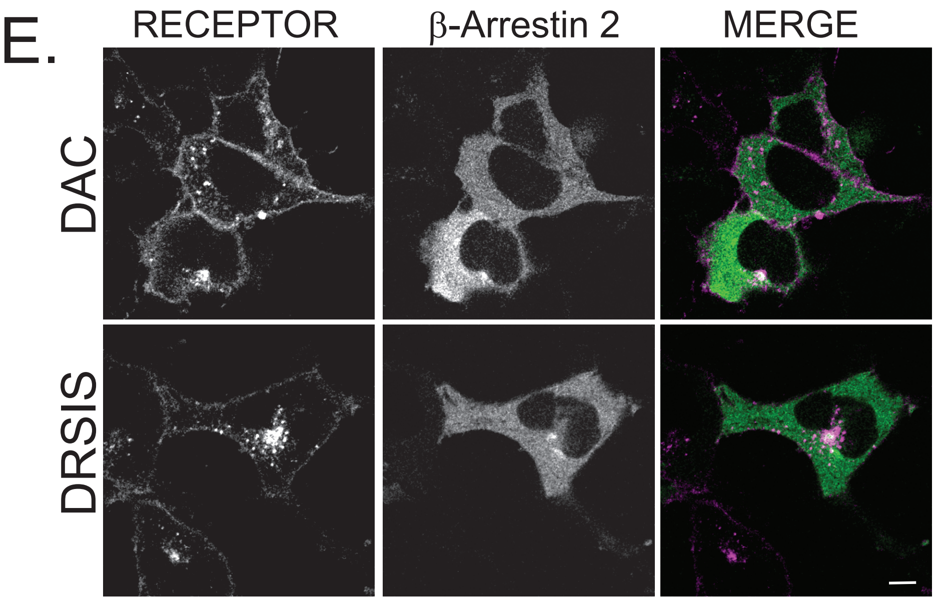

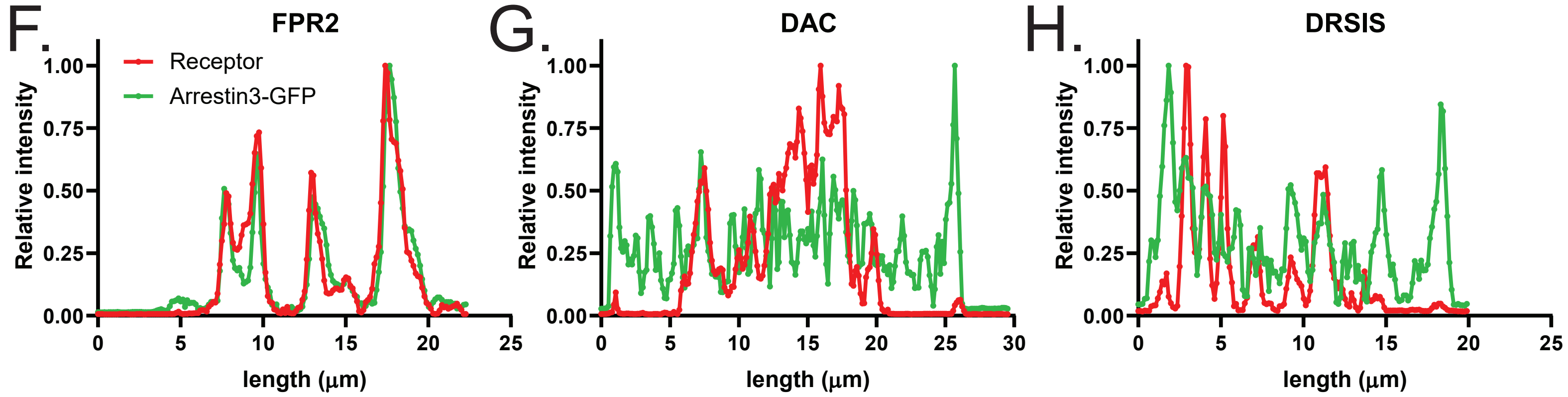

Supplemental Figure S7

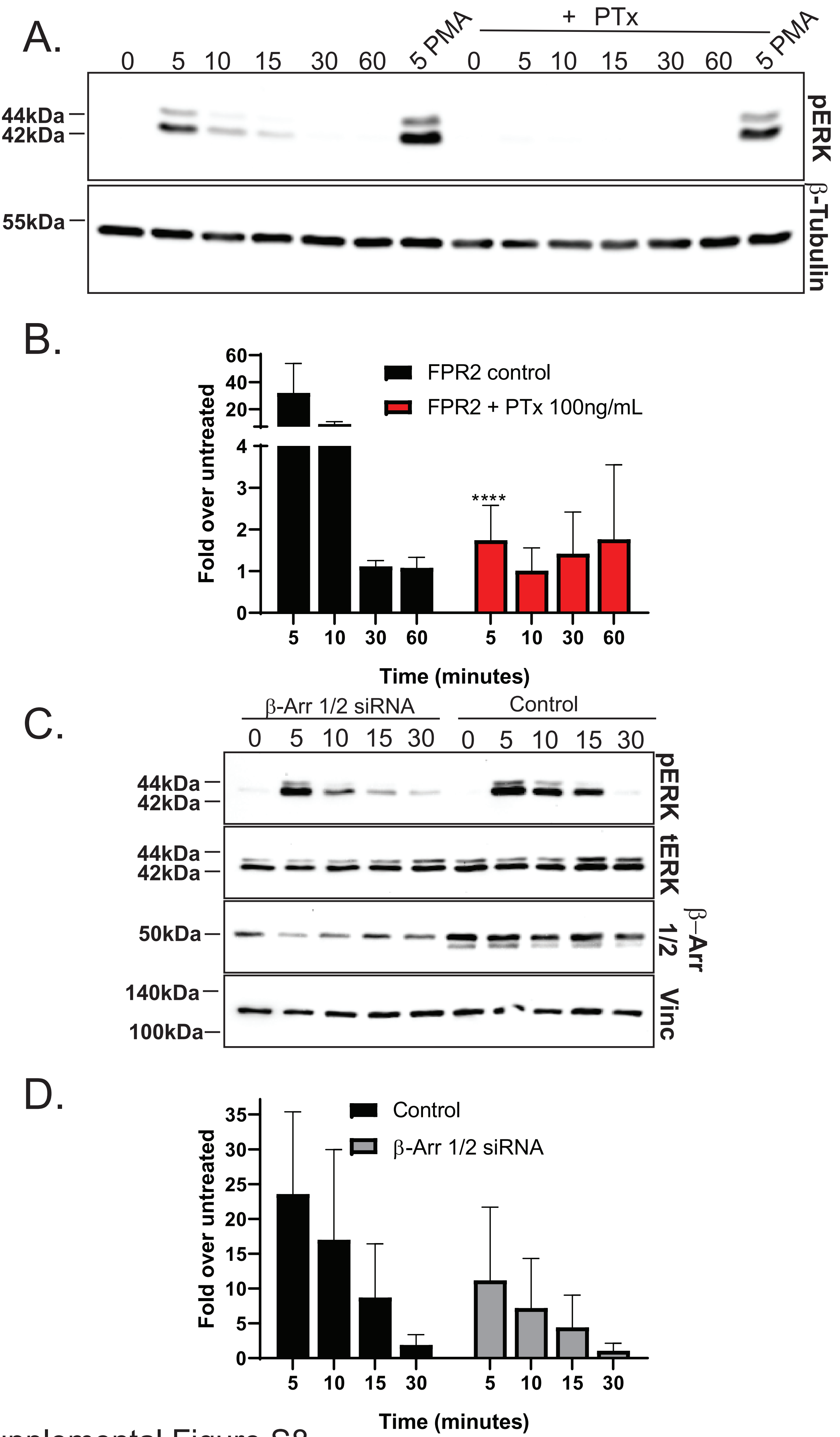

Supplemental Figure S8
